# SLC26A4-AP-2 mu2 interaction regulates SLC26A4 plasma membrane abundance in the endolymphatic sac

**DOI:** 10.1126/sciadv.adm8663

**Published:** 2024-10-09

**Authors:** Hyun Jae Lee, Cristina Fenollar-Ferrer, Kevin Isgrig, Ya-Xian Wang, Kerstin Valente, Juleh Eide, Keiji Honda, Wade W. Chien, Ronald S. Petralia, Lijin Dong, Thomas B. Friedman, Juan S. Bonifacino, Andrew J. Griffith, Isabelle Roux

**Affiliations:** ^1^ Otolaryngology Branch, National Institute on Deafness and Other Communication Disorders, National Institutes of Health, Bethesda, MD, USA.; ^2^ Laboratory of Molecular Genetics, National Institute on Deafness and Other Communication Disorders, National Institutes of Health, Bethesda, MD, USA.; ^3^ Inner Ear Gene Therapy Program, Neurotology Branch, National Institute on Deafness and Other Communication Disorders, National Institutes of Health, Bethesda, MD, USA.; ^4^ Advanced Imaging Core, National Institute on Deafness and Other Communication Disorders, National Institutes of Health, Bethesda, MD, USA.; ^5^ Department of Otorhinolaryngology, Tokyo Medical and Dental University, Bunkyo-ku, Tokyo, Japan.; ^6^ Department of Otolaryngology-Head and Neck Surgery, Johns Hopkins School of Medicine, Baltimore, MD, USA.; ^7^ Genetic Engineering Core, National Eye Institute, National Institutes of Health, Bethesda, MD, USA.; ^8^ Neurosciences and Cellular and Structural Biology Division, Eunice Kennedy Shriver National Institute of Child Health and Human Development, National Institutes of Health, Bethesda, MD, USA.; ^9^ Department of Otolaryngology, College of Medicine, University of Tennessee Health Science Center, Memphis, TN, USA.

## Abstract

Decreased presence or activity of human SLC26A4 at the plasma membrane is a common cause of hearing loss. SLC26A4 (Pendrin) is necessary for normal reabsorption of endolymph, the fluid bathing the inner ear. We identified the μ2 subunit of adaptor protein 2 (AP-2) complex required for clathrin-mediated endocytosis as a protein-partner of SLC26A4 involved in regulating its plasma membrane abundance. We showed that, in the endolymphatic sac, where fluid reabsorption occurs, SLC26A4 is localized along the apical microvilli of mitochondria-rich cells, in contact with the endolymph, and associated with clathrin-coated pits where μ2 and AP-2 are present. Based on SLC26A4 structure, the elements involved in SLC26A4-μ2 interaction were identified and validated experimentally, allowing modeling of this interaction at the atomic level. Pharmacological inhibition of clathrin-mediated endocytosis led to an increased plasma membrane abundance of hemagglutinin-tagged SLC26A4 virally or endogenously expressed in mitochondria-rich cells. These results indicate that the SLC26A4-μ2 interaction regulates SLC26A4 abundance at the apical surface of mitochondria-rich cells.

## Teaser

SLC26A4-μ2 interaction regulates SLC26A4 abundance at the apical surface of mitochondria-rich cells in the inner ear.

## INTRODUCTION

**Fig. 1.**
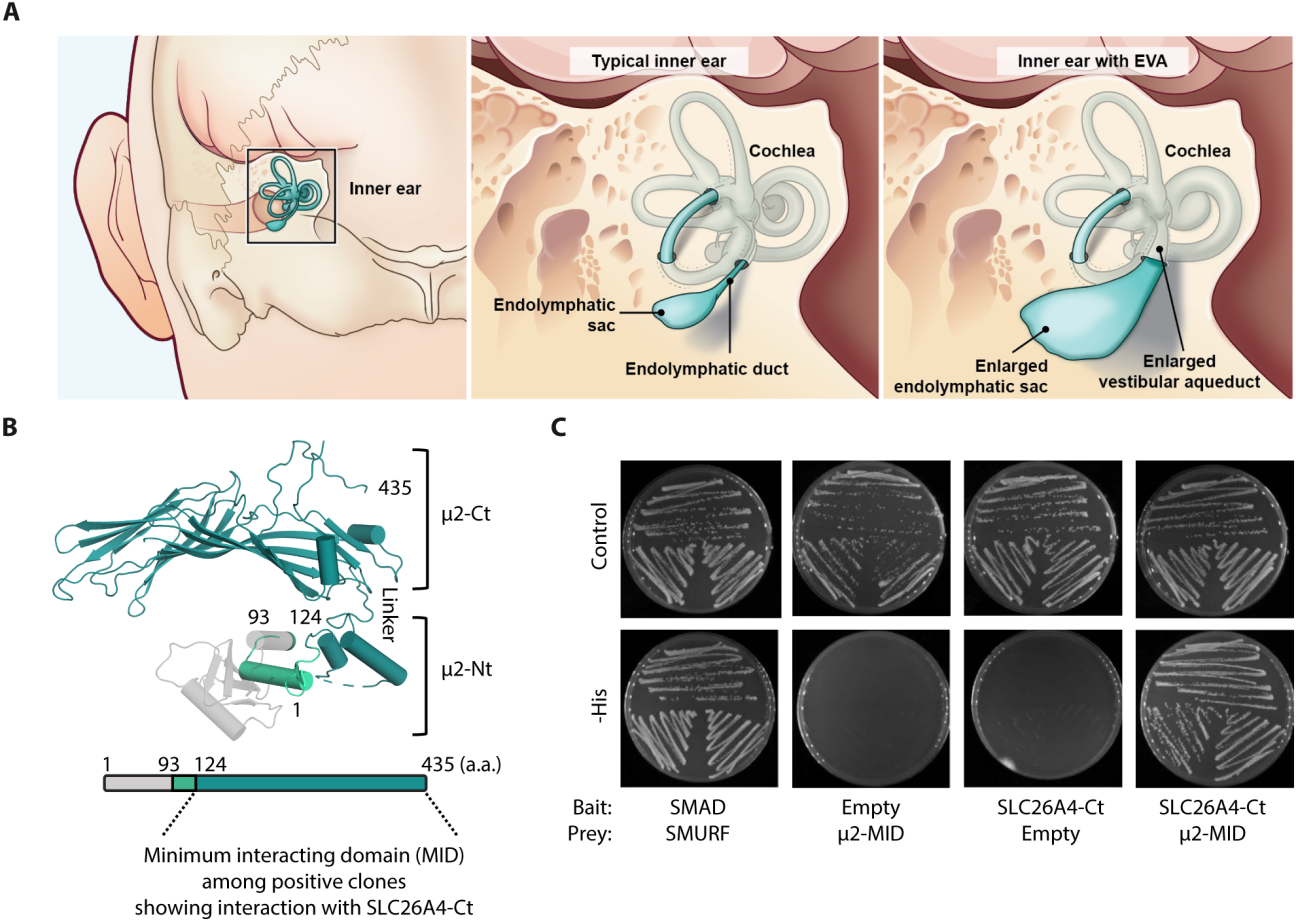
Schematic illustration of an enlarged vestibular aqueduct and endolymphatic sac and duct in human, and identification of the μ2 subunit of AP-2 as an interacting protein of SLC26A4. (**A**) Illustration of typical inner ear anatomy, endolymphatic duct and sac and, by comparison, EVA and endolymphatic sac [adapted from (*1*) and https://www.nidcd.nih.gov/health/enlarged-vestibular-aqueducts-and-childhood-hearing-loss]. (**B**) Structure of μ2 N-terminal (μ2-Nt) and C-terminal (μ2-Ct) domains. Five prey clones coding for part of μ2 were obtained from the kidney cDNA library screened with the C-terminal region of SLC26A4 (SLC26A4-Ct, residues 512 to 780). Two clones contained fragments of μ2 corresponding to residues 93 to 435, and three contained fragments corresponding to residues 124 to 435 of μ2, the MID (μ2-MID) among the interacting prey fragments identified in this screen. a.a., amino acid. (**C**) Results of Y2H assays showing the interaction of the SLC26A4-Ct bait and μ2-MID prey. Yeast expressing the bait and prey were grown in parallel on nonselective (Control) and selective without Histidine (-His) media. Yeast growth on plates without Histidine indicates the bait and prey interaction as observed with SMAD and SMURF, two proteins known to interact (positive control). No growth was seen when an empty vector replaced the vector expressing SLC26A4-Ct or μ2-MID (negative controls). *N* = 3.

Enlargement of the vestibular aqueduct (EVA) is a common morphological malformation of the inner ear detected in children with sensorineural hearing loss (HL) (*1*). In most cases, EVA is thought to result from enlargement of the endolymphatic sac and duct that runs through the vestibular aqueduct (Fig. 1A). The lumen of the endolymphatic sac contains a fluid called endolymph and is lined by a monolayer epithelium composed of mitochondria-rich cells (MRCs) and ribosome-rich cells (RRCs). MRCs are characterized by an abundance of mitochondria and numerous apical microvilli protruding into this lumen. RRCs have plentiful cytoplasmic ribosomes, rough endoplasmic reticulum, and cytoskeletal elements, in particular microtubules (*2*). Single-cell transcriptomic data suggest that MRCs play a role in endolymph ionic homeostasis and reabsorption, whereas RRCs are involved in protein synthesis, secretion, and innate immunity (*3*).

The *SLC26* gene family encodes transmembrane anion exchangers and anion channels. *SLC26A4* encodes SLC26A4 (aka pendrin), a polytopic transmembrane protein expressed predominantly in the inner ear, thyroid, and kidney (*4*). Pathogenic variants in *SLC26A4* are a common cause of HL associated with EVA, causing either nonsyndromic HL, DFNB4, or Pendred syndrome (association of HL and goiter due to an iodine organification defect), which both have a recessive inheritance (*5*, *6*). The cryo–electron microscopy (cryo-EM) structures of *Mus musculus* SLC26A4 show that SLC26A4 is a domain-swapped homodimer (*7*). Each protomer contains 14 transmembrane helices that form a central binding site, with intracellular N and C termini, the latter including a Sulfate Transporter–Anti-Sigma (STAS) factor antagonist domain. The STAS domain of one protomer interacts with the N-terminal domain of the other protomer and vice versa.

In the inner ear, SLC26A4 functions as a Cl^−^/HCO_3_^−^ anion exchanger (*8*). Light microscopy analyses have shown that SLC26A4 is expressed in the MRCs of the endolymphatic sac, where it is enriched in the apical region of the cells contacting the endolymph (*3*, *9*–*11*). SLC26A4 expression in the endolymphatic sac from embryonic day 16.5 (E16.5) to postnatal day 2 (P2) is required for normal structural and functional development of the mouse auditory system (*10*, *12*). SLC26A4 is necessary for normal endolymph reabsorption in the developing mouse endolymphatic sac, suggesting that reduced fluid reabsorption is the root cause of enlargement of the endolymphatic sac and duct observed in the absence of SLC26A4 (*3*).

Cells can regulate ion transport across their plasma membrane by modulating the levels and turnover of transmembrane transporters, channels, or ionic pumps at their cell surface (*13*). Clathrin-mediated endocytosis (CME) is a key process for controlling the abundance of a wide range of transmembrane proteins (“cargos”), including ion channels, transporters, and G protein–coupled receptors at the cell surface (*14*). Cargo proteins are recruited into clathrin-coated pits (CCPs), which are pinched off from the plasma membrane in a dynamin-dependent mechanism to form cytoplasmic clathrin-coated vesicles (CCVs) (*15*–*17*). The adaptor protein 2 (AP-2) complex acts as an adaptor between the cargo and the clathrin lattice during internalization. This heterotetrameric complex is formed by two large (α and β2), a medium (μ2), and a small (σ2) subunit. The μ2 subunit has been implicated in the recruitment of cargos into CCPs through recognition of a tyrosine-based motif (*18*–*22*).

This study provides insight into the regulation of SLC26A4 in the inner ear by identifying the μ2 subunit of AP-2 as an interacting protein that regulates SLC26A4 abundance at the plasma membrane of MRCs through CME. Using the cryo-EM structures of SLC26A4 (*7*), the structural elements involved in this interaction were identified and validated experimentally, allowing the modeling at the atomic level of the mouse and human SLC26A4-μ2 complexes. Pharmacological inhibition of CME increased the amount of SLC26A4 at the apical plasma membrane of the endolymphatic sac epithelium available for exchange of Cl^−^ and HCO_3_^−^.

## RESULTS

### The μ2 subunit of the AP-2 complex interacts with SLC26A4

**Fig. 2.**
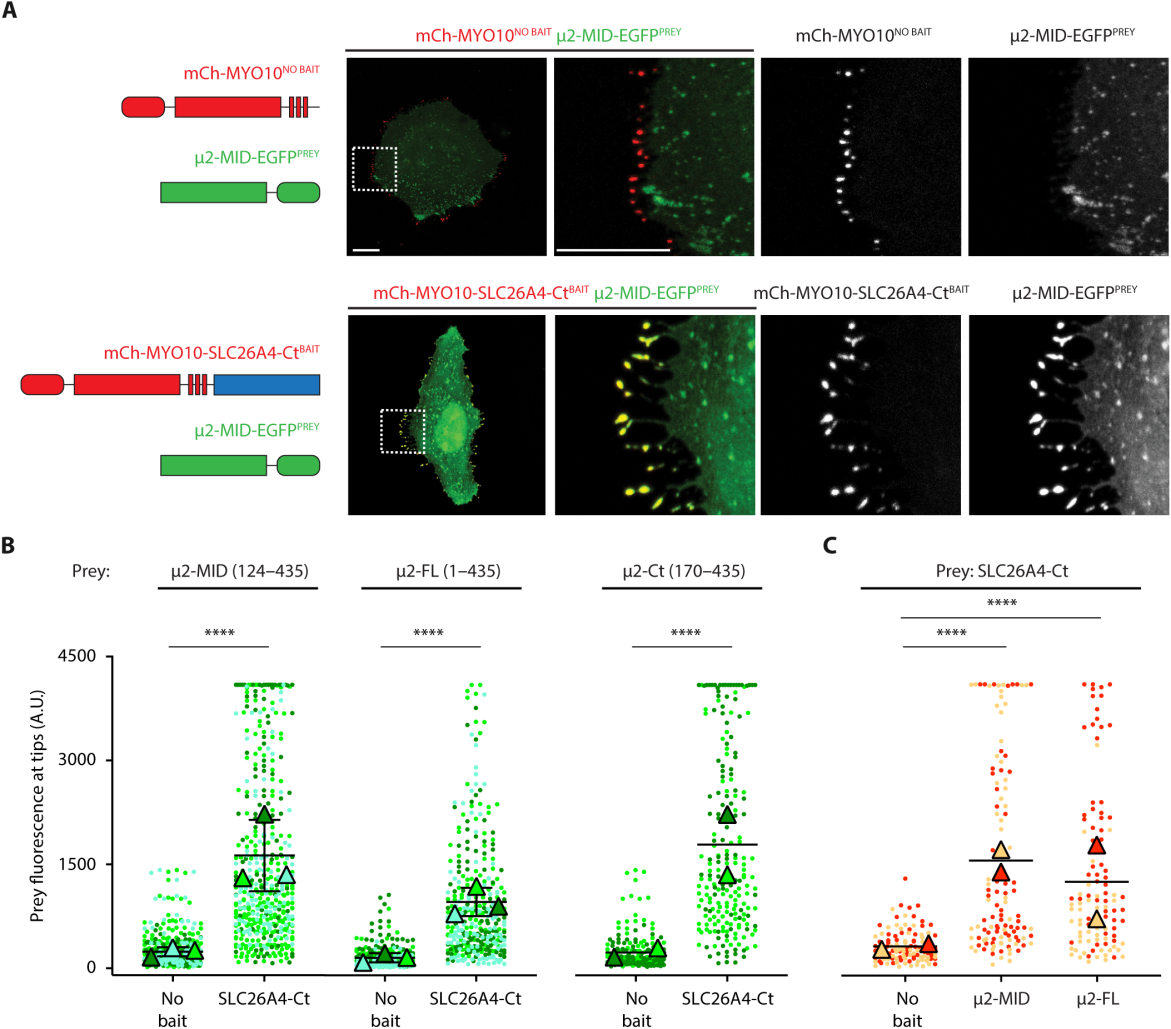
NanoSPD assays validating the interaction of SLC26A4 with μ2 in HeLa cells. (**A**) When coexpressed in HeLa cells, mCherry-MYO10^NO BAIT^ (mCh-MYO10^NO BAIT^) was enriched at filopodia tips (red), whereas μ2 MID-EGFP^PREY^ was detected in the cytoplasm without enrichment at filopodia tips (green) (upper panels). In contrast, when μ2 MID-EGFP^PREY^ (green) was coexpressed with mCherry-MYO10-SLC26A4-Ct (mCh-MYO10-SLC26A4-Ct^BAIT^) (red), both proteins were enriched at filopodia tips (yellow) (lower panels). Together, these results indicated that SLC26A4-Ct can recruit μ2-MID to filopodia tips. (**B**) Quantification of the fluorescence of μ2-MID-EGFP^PREY^, μ2 full-length (FL)-EGFP^PREY^, or μ2 C-terminal region (Ct)-EGFP^PREY^ at filopodia tips when coexpressed with mCh-MYO10^NO BAIT^ or mCh-MYO10-SLC26A4-Ct^BAIT^. (**C**) Quantification of reciprocal experiments. mCh-SLC26A4-Ct^PREY^ was enriched at filopodia tips when coexpressed with EGFP-MYO10-μ2-MID^BAIT^ or EGFP-MYO10-μ2-FL^BAIT^ but not when coexpressed with EGFP-MYO10^NO BAIT^. For each condition, prey fluorescence at filopodia tips from 106 to 439 filopodia is shown. Each dot corresponds to the fluorescence quantified at one filopodium tip and was color coded by biological replicate. Triangles show the mean value per biological replicate. The mean ± SD of these means is shown when *N* > 2. Experiments were quantified blind to the transfected plasmids. *t* test (B) or one-way ANOVA followed by Tukey’s multiple comparison tests (C) were used to compare results under different conditions. The value for each filopodium was considered as an independent measurement. ****P < 0.0001. Scale bars, 10 μm.

To identify proteins that interact with SLC26A4, the cytosolic C-terminal region of mouse SLC26A4 (SLC26A4-Ct, NP_035997.1, residues 512 to 780) was used as the bait to screen a yeast two-hybrid (Y2H) library constructed with cDNA derived from 8- to 10-week-old mouse kidneys. This Y2H prey library was chosen because SLC26A4 is highly expressed in type B and non-A, non-B intercalated cells of the renal collecting duct (*23*, *24*). We hypothesized that some partners of SLC26A4 important for its regulation and function in inner ear MRCs may also be expressed in renal cells, which are also involved in fluid reabsorption (*23*–*25*). Five prey clones, at least two of which were independent, encoded the C-terminal region of the μ2 subunit of the AP-2 complex (Fig. 1B). Their overlapping amino acid sequence defined a minimum interacting domain (MID) (NP_033809.1, residues 124 to 435). To confirm this interaction, we used Y2H pairwise assays with SLC26A4-Ct as the bait and μ2-MID as the prey. Yeast cells expressing both constructs grew on selective media, but yeast cells transfected with only one of these vectors and a control empty vector did not grow, confirming the interaction of SLC26A4-Ct with μ2-MID in yeast cells (Fig. 1C). To also validate this interaction in a mammalian system, we used nanoscale pulldown (NanoSPD) assays based on the ability of the myosin-10 heavy meromyosin-like domain (MYO10^HMM^ designated here as MYO10) to move along F-actin and accumulate at filopodia distal tips of mammalian cells (*26*). By expressing our bait protein fused with MYO10 in HeLa cells, it can be transported and accumulates at the filopodia tips. If the prey protein also accumulates at the filopodia tips when coexpressed with the bait, while it is diffuse in the cytoplasm in cells expressing MYO10 without the bait, then it is indicative of bait and prey interaction. SLC26A4-Ct was fused to the C-terminal region of mCherry-tagged MYO10 (mCh-MYO10-SLC26A4-Ct) and used as the bait. The prey protein was obtained by fusing enhanced green fluorescent protein (EGFP) to the C terminus of the MID of μ2 (μ2-MID-EGFP) (table S1). When μ2-MID-EGFP was coexpressed with mCh-MYO10-SLC26A4-Ct in HeLa cells, it showed robust enrichment at filopodia tips (Fig. 2, A and B). In contrast, coexpression with mCh-MYO10^NO BAIT^ (negative control) did not result in enrichment of green fluorescence at filopodia tips (Fig. 2, A and B), indicating that μ2-MID was cotransported via its interaction with SLC26A4-Ct. Similarly, EGFP fused to either full-length μ2 (μ2-FL, residues 1 to 435) or its C-terminal domain (μ2-Ct, residues 170 to 435) showed a significant enrichment at filopodia tips when coexpressed with mCh-MYO10-SLC26A4-Ct (Fig. 2B). Reciprocally, mCh-SLC26A4-Ct was enriched at filopodia tips when coexpressed with EGFP-MYO10-μ2-MID or EGFP-MYO10-μ2-FL but not when it was coexpressed with EGFP-MYO10^NO BAIT^ (Fig. 2C). Together, these results indicate that the μ2 subunit of the clathrin adaptor AP-2 complex can interact with SLC26A4-Ct in yeast and human cultured cells.

### SLC26A4 and AP-2 are colocalized in the endolymphatic sac

**Fig. 3.**
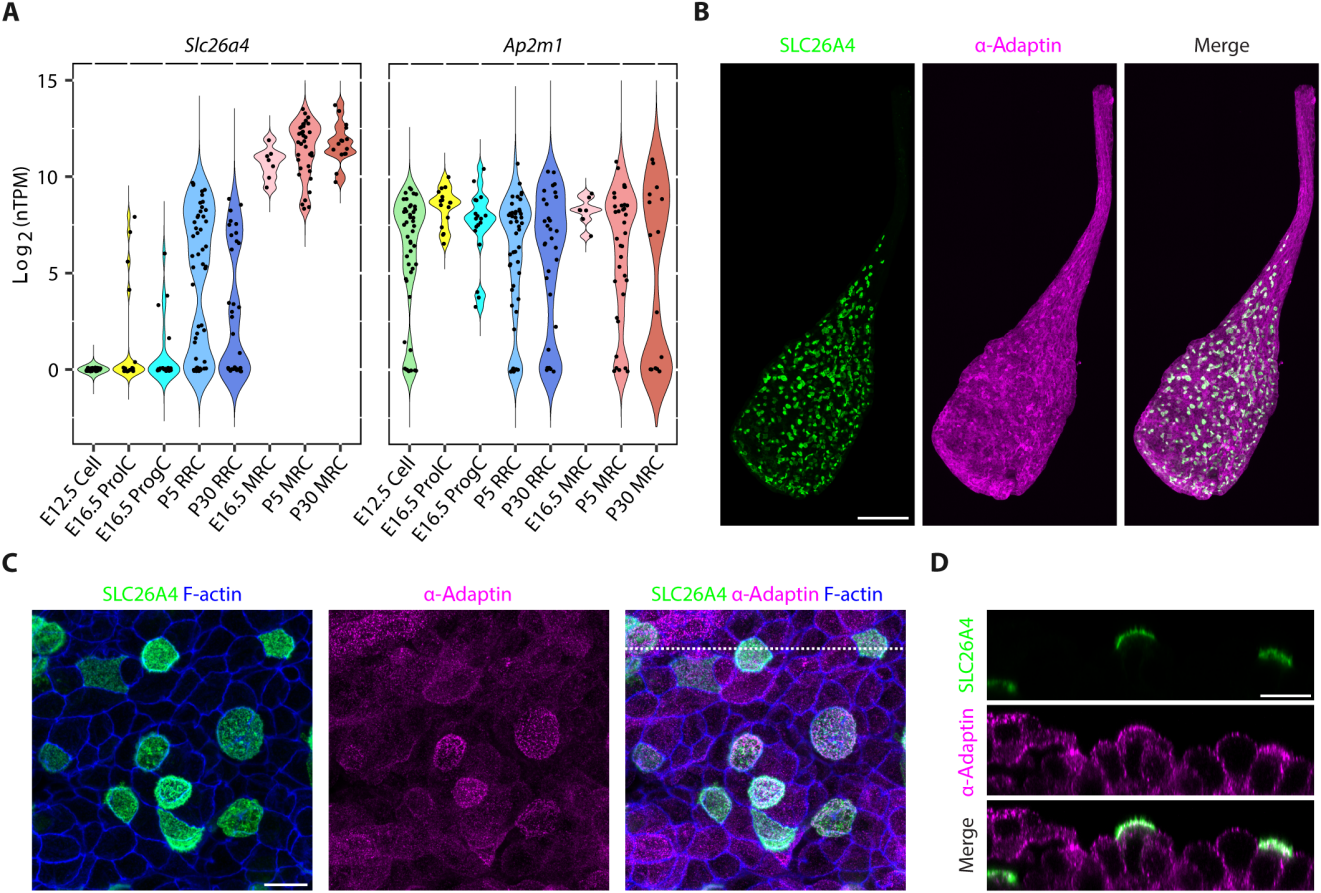
SLC26A4 and AP-2 expression and localization in the endolymphatic sac. (**A**) *Slc26a4* and *Ap2m1* transcript levels in cells of developing mouse endolymphatic sac (ProlC, proliferating cell; ProgC, progenitor cell; RRC, ribosome-rich cell; MRC, mitochondria-rich cell) at different ages [scRNA-seq data (*3*)]. *Slc26a4* is highly expressed in MRCs at E16.5, P5, and P30. *Ap2m1* is expressed in all cell types at all time points studied. In these violin plots, black dots indicate the expression level of the gene as log_2_ normalized transcripts per million (nTPM) for a cell. (**B** to **D**) Confocal images of whole-mount E16.5 endolymphatic sac immunolabeled for SLC26A4 and α-adaptin used as a proxy of AP-2. Representative maximum intensity projection images of whole endolymphatic sac (B). Higher-magnification images of opened endolymphatic sac (C). Reconstructed tissue cross section at the level of the white dotted line in (C) shows that SLC26A4 and α-adaptin are enriched at the apical luminal membrane of MRCs (D). No qualitative difference was observed between labeling in tissues from male and female mice. Scale bars, 100 μm (B) and 10 μm [(C) and (D)].

To examine if *Slc26a4* and *Ap2m1*, encoding μ2, are coexpressed within endolymphatic sac cells, we analyzed single-cell transcriptomic data from C57BL/6J mouse endolymphatic sac epithelia (*3*). *Ap2m1* transcripts were detected in all cell types identified in the endolymphatic sac at E12.5, E16.5, P5, and P30, including the MRCs, which express *Slc26a4* (Fig. 3A). To determine if μ2 and AP-2 colocalize with SLC26A4 in MRCs, we compared the distributions of the endogenous proteins in the E16.5 mouse endolymphatic sac. Anti–α-adaptin antibodies, which recognize the AP-2 α subunit, were used as a proxy to localize the AP-2 complex as there are no μ2-specific antibodies suitable for immunofluorescence microscopy. As described (*3*, *9*, *11*), SLC26A4 was enriched at or near the apical surface of the MRCs (Fig. 3, B to D). Anti–α-adaptin immunoreactivity had a punctate pattern in all cells of the endolymphatic sac and duct. However, a higher density of labeling was present in cells expressing SLC26A4. It was enriched in the apical region of cells where SLC26A4 immunolabeling was also concentrated (Fig. 3, B to D). This enrichment and colocalization of SLC26A4 and α-adaptin in the apical region of the MRCs support our hypothesis of a physiological interaction between SLC26A4 and the AP-2 complex, via its μ2 subunit.

### SLC26A4 is present along microvilli of MRCs and associated with CCPs and CCVs

**Fig. 4.**
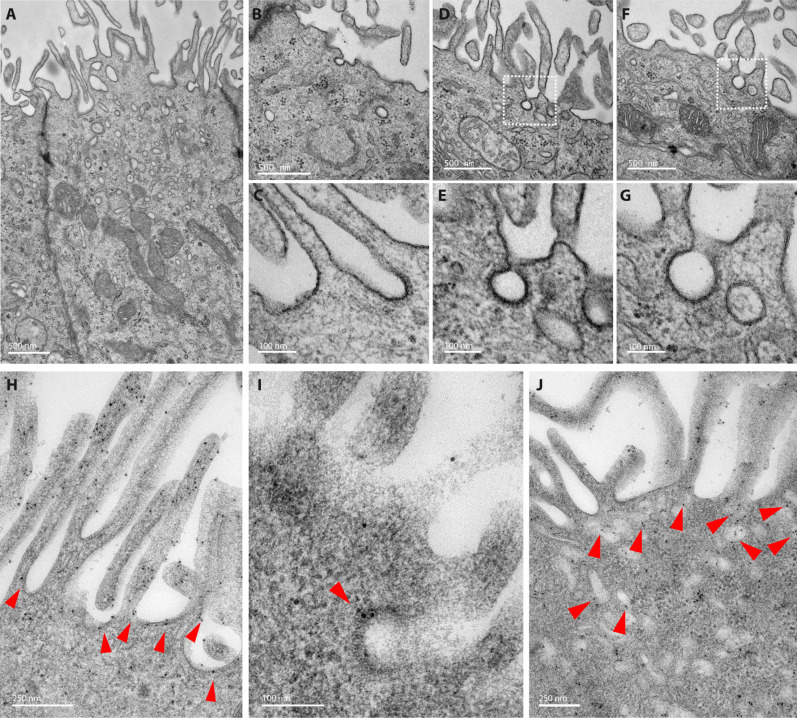
Ultrastructural characteristics of the MRCs of the mouse endolymphatic sac and subcellular localization of SLC26A4. (**A** to **G**) TEM images of MRCs of P0 mouse endolymphatic sac showing numerous apical microvilli, mitochondria, and intracellular vesicles. CCPs at the base of two microvilli are seen in (C), (D), and (F) [(D) and (F) also shown at a higher magnification in (E) and (G)]. (**H** to **J**) Immunogold EM images of endolymphatic sac labeled with anti-SLC26A4 antibodies and secondary antibodies coupled to gold particles. SLC26A4 labeling is concentrated along the microvilli (H) but is also present at their base (red arrows) and associated with CCPs (I) and detected in a subset of the many vesicles present in the MRC apical region (J). Similar results were obtained in samples from males and females. Scale bars, 500 nm [(A), (B), (D), and (F)], 250 nm [(H) and (J)], and 100 nm [(C), (E), (G), and (I)].

To study MRCs and the subcellular distribution of SLC26A4 in these cells, previously only studied by confocal microscopy (*3*, *10*), we used transmission electron microscopy (TEM) and immunogold TEM. The MRCs of P0 and P5 C57BL/6J mice have numerous mitochondria, rounded nuclei, and an abundance of microvilli protruding into the lumen of the endolymphatic sac (Fig. 4A), similar to adult rat MRCs (*2*, *27*). We observed CCPs at the base of microvilli and a large number of vesicles of various sizes in the apical region of these cells (Fig. 4, A to G). We detected tight junctions between MRCs and neighboring cells (Fig. 4A) as previously described (*28*). SLC26A4 ultrastructural localization was studied at P0 using antibodies that recognize SLC26A4-Ct [PB826 (*3*, *10*)]. SLC26A4 was detected along the microvilli where a high density of immunogold particles was present (Fig. 4H and fig. S1). SLC26A4 was also detected in CCPs (Fig. 4I) and in the vesicles accumulated in the apical region of MRCs (Fig. 4J). These data suggest that CME is involved in the endocytosis of SLC26A4 from the apical surface of the MRCs and reveal the presence of SLC26A4 in a pool of apical vesicles in MRCs. One hypothesis is that at least some of these apical vesicles are a source of SLC26A4 that can either replenish the pool of SLC26A4 present at the plasma membrane or contribute to the modulation of its abundance in response to changes in cell surface tension or ionic composition of the endolymph.

### SLC26A4 interacts with AP-2 μ2 via the SLC26A4 536-YKNL tyrosine-based motif

**Fig. 5.**
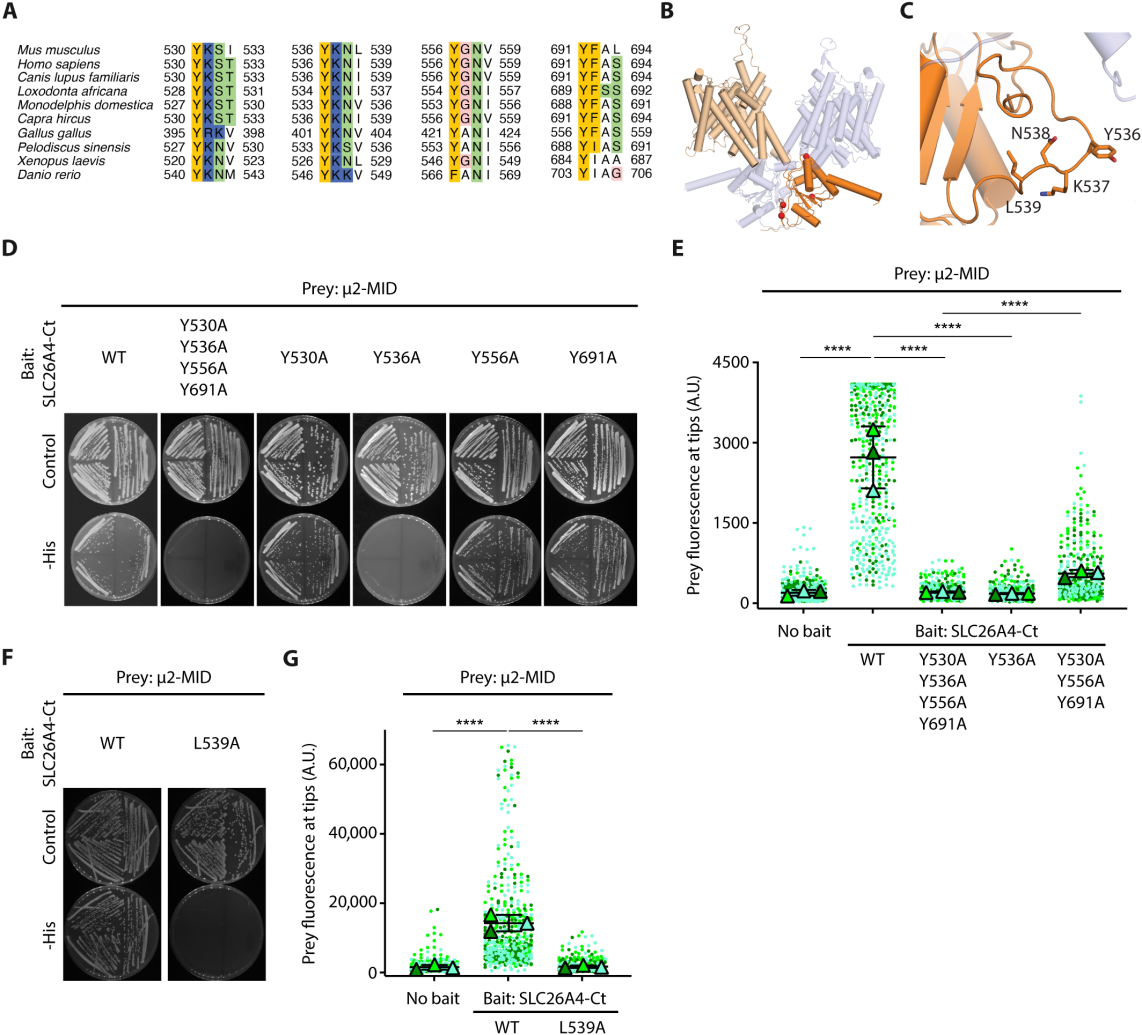
Identification of a tyrosine-based motif in the cytosolic C-terminal domain of SLC26A4 necessary for its interaction with μ2. (**A**) Conservation across different species of the residues forming the four tyrosine-based motifs present in mouse SLC26A4-Ct. Residues are colored according to their physicochemical properties. (**B**) Cryo-EM structure of the mouse SLC26A4 dimer in asymmetric form (PDB ID: 7wk1) (*7*). Transmembrane and STAS domains of one monomer are highlighted in light and dark orange, respectively. Each red sphere represents the Cα atom of the tyrosine of each of these motifs. (**C**) Close-up view. Only 536-YKNL is in a flexible loop partially exposed to the solvent. (**D**) Interaction of μ2-MID with SLC26A4-Ct, either WT or carrying a tyrosine to alanine substitution at the different tyrosine-based motifs was tested (Y530A, Y536A, Y556A, and Y691A) in Y2H assays. Y536 is necessary for SLC26A4-Ct-μ2-MID interaction. *N* = 3. (**E**) Quantification of NanoSPD assays showing the fluorescence of μ2-MID-EGFP^PREY^ at filopodia tips when coexpressed in HeLa cells with mCh-MYO10^NO BAIT^ or mCh-MYO10-SLC26A4-Ct^BAIT^, either WT or carrying different combinations of substitutions affecting Y530, Y536, Y556, and Y691. (**F**) Y2H assay results showing that L539 is necessary for the SLC26A4-Ct-μ2-MID interaction. *N* = 3. (**G**) Quantification of NanoSPD assays showing a similar result. [(E) and (G)] For each condition, prey fluorescence at filopodia tips from 344 to 431 filopodia is shown. Each dot corresponds to the fluorescence quantified at one filopodium tip and was color coded by biological replicate. Triangles show the mean value per biological replicate. The mean ± SD of these means is shown. One-way ANOVA followed by Tukey’s multiple comparison tests were used to compare results under different conditions. The value for each filopodium was considered as an independent measurement. ****P < 0.0001.

To identify the structural elements involved in the SLC26A4-μ2 interaction, we sought the presence of canonical tyrosine-based motifs (YXXΦ, X being any residue and Φ a bulky hydrophobic residue) previously found in the proteins binding μ2 (*29*–*31*) in SLC26A4-Ct. Mouse SLC26A4-Ct contains four such motifs starting at positions 530, 536, 556, and 691 (NP_035997.1), while the corresponding region in human SLC26A4 only contains two, at positions 536 and 556 (NP_000432.1). These two motifs are the most phylogenetically conserved, whereas motifs starting at positions 530 and 691 do not show a conserved fourth bulky hydrophobic residue (Fig. 5A).

We used the recently solved cryo-EM structures of mouse SLC26A4 (*7*) to determine the structural localization of these motifs and their potential availability to interact with μ2 (Fig. 5B). SLC26A4 structures, independently of their conformation, showed that the residues of the motif starting at position 556 interact with neighboring residues in the core of the protein. In contrast, at least two residues in the motif starting at position 536 are exposed to the solvent and only Tyr^536^ (Y536) is involved in intramolecular interactions with the adjacent helix. This motif is located within a highly flexible intracellular loop exposed to the solvent that could easily expose Y536 when the interacting partner approaches. This tyrosine-based motif has a similar configuration to the tyrosine-based motif of human insulin receptor substrate 1 (IRS1) bound to the human μ2 subunit in the x-ray structure [Protein Data Bank (PDB) ID: 6bnt (*29*)], suggesting that SLC26A4 could interact with μ2 using a similar region to that identified in the IRS1. These results predicted that the tyrosine-based motif 536-YKNL in mouse SLC26A4-Ct (536-YKNI in human SLC26A4) is the most likely region for SLC26A4 to interact with μ2 (Fig. 5, B and C).

To test this prediction experimentally, site-directed mutagenesis was used to substitute alanine for the tyrosine residues of the four motifs identified in mouse SLC26A4. We used Y2H assays to compare the interaction of μ2-MID with wild-type (WT) SLC26A4-Ct, SLC26A4-Ct with all four tyrosine-based motifs mutated, and SLC26A4-Ct with each of the tyrosine residues mutated individually. As shown by the differences in yeast growth (Fig. 5D), the interaction between SLC26A4-Ct and μ2-MID was lost when SLC26A4-Ct was mutated at all four motifs (Y530A, Y536A, Y556A, and Y691A) or at Y536A alone, whereas it was maintained when the other single tyrosine residues were substituted for alanine at position 530, 556, or 691 (Y530A/Y556A/Y691A). These results implicate Y536 as a necessary residue required for interaction with the μ2 subunit in yeast cells. Using a similar approach with NanoSPD assays, we tested if these different mutations could interfere with the interaction of mCh-MYO10-SLC26A4-Ct with μ2-MID-EGFP in HeLa cells. SLC26A4-Ct with all four Y530A, Y536A, Y556A, and Y691A substitutions or with the single Y536A substitution did not show interaction with μ2 (Fig. 5E), further supporting the essential role of Y536 for the SLC26A4-Ct-μ2-MID interaction. The interaction was partially rescued when Y536 was left intact, and the three other tyrosine residues were mutated to alanine. Although this result may suggest a contribution of one or more of the other motifs (Y530, Y556, and Y691) to the interaction, it may also reflect the structural changes and possible unfolding in SLC26A4 carrying these substitutions that interferes with the ability of the Y536 motif to interact with μ2. We also tested if reducing the hydrophobicity of the fourth residue of the 536-YKNL motif, L539, could affect the interaction of SLC26A4-Ct with μ2-MID. In both Y2H and NanoSPD assays, the L539A substitution led to a loss of the interaction between the mutated SLC26A4-Ct and μ2-MID (Fig. 5, F and G). These results are consistent with the key role of the hydrophobic residue L539 in the 536-YKNL motif for the SLC26A4-Ct-μ2-MID interaction. Together, these results support the hypothesis that the tyrosine-based motif 536-YKNL in SLC26A4-Ct is necessary, if not entirely sufficient, for the interaction of SLC26A4-Ct with μ2.

### μ2 residues F174, D176, and W421 are involved in the interaction with SLC26A4

**Fig. 6.**
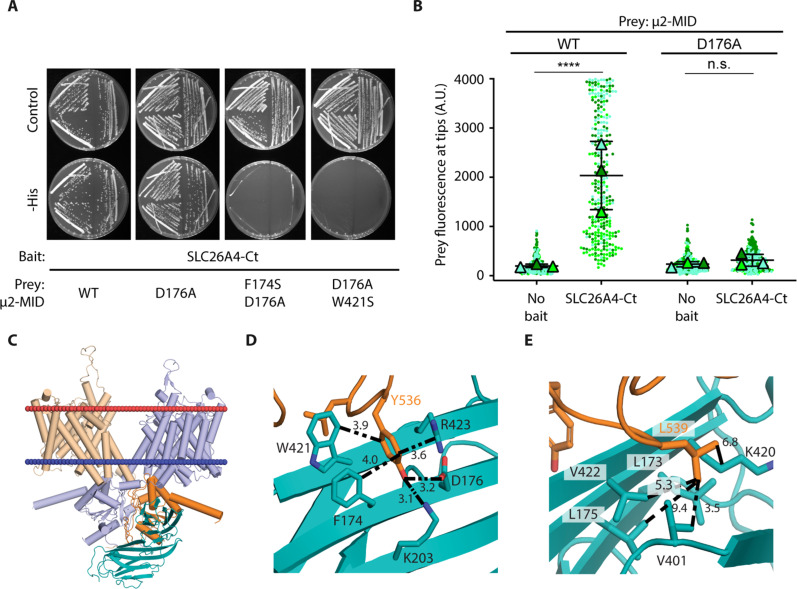
Residues in the binding pocket of μ2 necessary for its interaction with SLC26A4. (**A**) Y2H assay results evaluating the interaction of SLC26A4-Ct and μ2-MID domain, either WT or carrying substitutions affecting different residues of its binding pocket (F174S, D176A, and W421S). *N* = 3. (**B**) Quantification of NanoSPD assays between SLC26A4-Ct and μ2-MID WT or with the substitution D176A. For each condition, prey fluorescence at filopodia tips from 323 to 349 filopodia is shown. Each dot corresponds to the fluorescence quantified at one filopodium tip and was color coded by biological replicate. Triangles show the mean value per biological replicate. The mean ± SD of these means is shown. One way ANOVA followed by Tukey’s multiple comparison tests were used to compare results under different conditions. The value for each filopodium was considered as an independent measurement. (**C**) The structural model of mouse SLC26A4 complexed with the μ2 subunit, obtained after the protein-protein docking protocol, is shown with the membrane represented by two sphere planes indicating extracellular (red) and intracellular (blue) lipid leaflets. SLC26A4 monomers are colored in orange and blue, while μ2 is colored in emerald-green. (**D** and **E**) Close-up views of binding sites in μ2 for Y536 and the hydrophobic residue L539 within the SLC26A4 tyrosine-based motif. Residues in μ2 coordinating with these two residues are shown as sticks, and their corresponding interactions are indicated as dashed lines; distances are presented in angstroms. Nitrogen and oxygen are colored in blue and red, respectively. ****P < 0.0001; n.s., not significant.

Tyrosine-based motifs of cargo proteins interact with μ2 through a binding pocket located at its C-terminal domain (*19*). The aromatic ring of the tyrosine residue of the motif has been described to have hydrophobic interactions with residues F174 and W421 of μ2. The binding of this tyrosine is further stabilized by the hydrogen bonds between its hydroxyl group and the side chains of μ2 residues D176, K203, and R423 (*19*). To test if this same binding pocket of μ2 is also involved in the interaction with SLC26A4-Ct, we performed Y2H assays using constructs of μ2-MID with different residue substitutions and tested their interaction with SLC26A4-Ct. The single substitution D176A was not sufficient to abolish the interaction of μ2-MID with SLC26A4-Ct in yeast cells. However, no interaction was detected in the presence of the double substitutions F174S/D176A or D176A/W421S (Fig. 6A).

Effects of changes in this binding pocket were also investigated using NanoSPD assays. μ2-MID, either WT or carrying the substitution D176A, was fused to EGFP and coexpressed with mCh-MYO10-SLC26A4-Ct in HeLa cells. Contrary to EGFP-μ2-MID WT, which was transported to the filopodia tips with mCh-MYO10-SLC26A4-Ct, EGFP-μ2-MID D176A remained diffuse in the cell cytoplasm (Fig. 6B). This result suggests that D176 in μ2 is necessary for the interaction of μ2-MID with SLC26A4-Ct in NanoSPD assays. As the mutation D176A was not sufficient to abolish the interaction of μ2-MID with SLC26A4-Ct in Y2H assays, this result highlights the different sensitivity of the two approaches we used and the benefit of conducting these parallel protein interaction assays. Together, these results show that the same tyrosine-binding pocket previously described for interactions of μ2 with other cargo molecules is involved in its interaction with SLC26A4.

### Structural characterization of the SLC26A4-μ2 interaction

The resolution of any given protein structure is highest in regions that are part of the core of the protein and are not flexible. On the contrary, most of the time, the configuration of loop regions shown in these structures only represents a snapshot of the myriad of possible configurations accessible to these inherently flexible segments and depends, mostly, on the crystallization conditions such as the solvent and the presence of other ions or solutes. Some other conformations might be available when the protein is in the cell membrane or the substrate approaches the targeted loop. To identify the most probable configuration of the loop containing the tyrosine-based motif 536-YKNL in SLC26A4 that optimizes its interaction with μ2, we performed loop remodeling of this region with MODELLER (*32*). The model with the loop configuration that resulted in the highest solvent accessible surface area (SASA) values, greater than 60% for the four residues of 536-YKNL (fig. S2), was selected as the input for the protein-protein docking simulations with μ2 (referred to as SLC26A4 loop 1 model). The maximum exposure of these residues ensures maximum probability to interact with μ2.

The mouse SLC26A4 loop 1 model was docked to the mouse μ2 protein structure obtained from the x-ray structure of human μ2 (residues 161 to 435) [PDB ID: 6bnt (*29*)] using the protein-protein docking procedure implemented in the HADDOCK server v2.2 (fig. S3) (*33*, *34*). The amino acid sequences of these μ2 orthologs share 100% sequence identity in this segment, implying that their structures are also identical. In addition to the canonical energy function and shape complementarity common with other protein-protein docking algorithms, HADDOCK also incorporates experimental data as restraints to guide the docking process increasing the efficiency of the algorithm, which, in some cases, produces complexes similar to those deposited in the PDB. These specific restraints can be introduced as additional terms in the energy function and can be either defined as distance restraints between two residues and/or as a set of active and passive residues within the two proteins of interest. Residues known to be necessary for the interaction to occur are defined as active and must be part of the interacting interface throughout the docking run. Residues that are in the proximity of the active residues and that can potentially partake in the interaction are defined as passive. Passive residues do not have to be part of the interacting interface, and so, contrary to active residues, no energy penalty is added to the scoring function if that is the case.

We thus analyzed the x-ray structure of the complex formed between a tyrosine-based motif and human μ2 and identified the residues in μ2 that establish the interaction with the tyrosine and hydrophobic residue in the tyrosine-binding motif and measured the distances between their corresponding Cα atoms (table S2). These distances were then used as distance restraints between the equivalent Y536 and L539 of mouse SLC26A4 and the corresponding interacting residues in μ2 to guide the docking process.

**Fig. 7.**
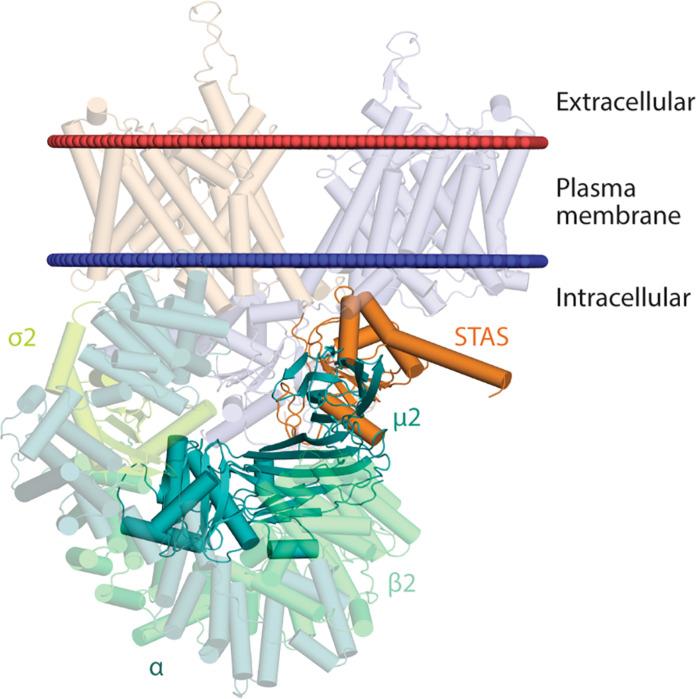
Structural model of mouse SLC26A4 complexed with AP-2. The structural model of mouse SLC26A4 in complex with AP-2 was obtained after structural superimposition of the μ2 subunit in the SLC26A4-μ2 complex and that in the x-ray structure of *Rattus norvegicus* AP-2 in open form [PDB ID: 2xa7 (*35*)]. SLC26A4 monomers are colored in orange and blue, while AP-2 subunits are colored in different shades of green.

In the complex we obtained after this docking protocol (Fig. 6C), Y536 of SLC26A4 establishes three hydrophobic interactions with the aliphatic chain of R423, the aromatic group of F174 and W421, a cation-π interaction with the guanidino group in R423 and two hydrogen bonds with the heteroatoms of D176 and K203 of μ2 (Fig. 6D). The hydrophobic residue L539 of SLC26A4 establishes three hydrophobic interactions with the side chains of residues V401, V422, and the aliphatic chain of K420 of μ2 (Fig. 6E). These two interacting networks are similar to those observed in the x-ray structures of a tyrosine peptide bound to μ2 (PDB ID: 6bnt) or AP-2 [PDB ID: 2xa7 (*35*)], indicating that 536-YKNL of SLC26A4 can interact with μ2 in a similar arrangement as those with canonical tyrosine-based motifs and that this tyrosine-based motif mediates the interaction between SLC26A4 and μ2 (Fig. 6, C to E). Similar binding was obtained for human SLC26A4 and human μ2 (sequence alignment used in fig. S4 and models obtained in figs. S5 and S6). Complexes between mouse and human SLC26A4 and their corresponding AP-2 partners are shown in Fig. 7 and fig. S7, respectively.

### HA-SLC26A4 presence at the plasma membrane is increased by pharmacological inhibition of CME

**Fig. 8.**
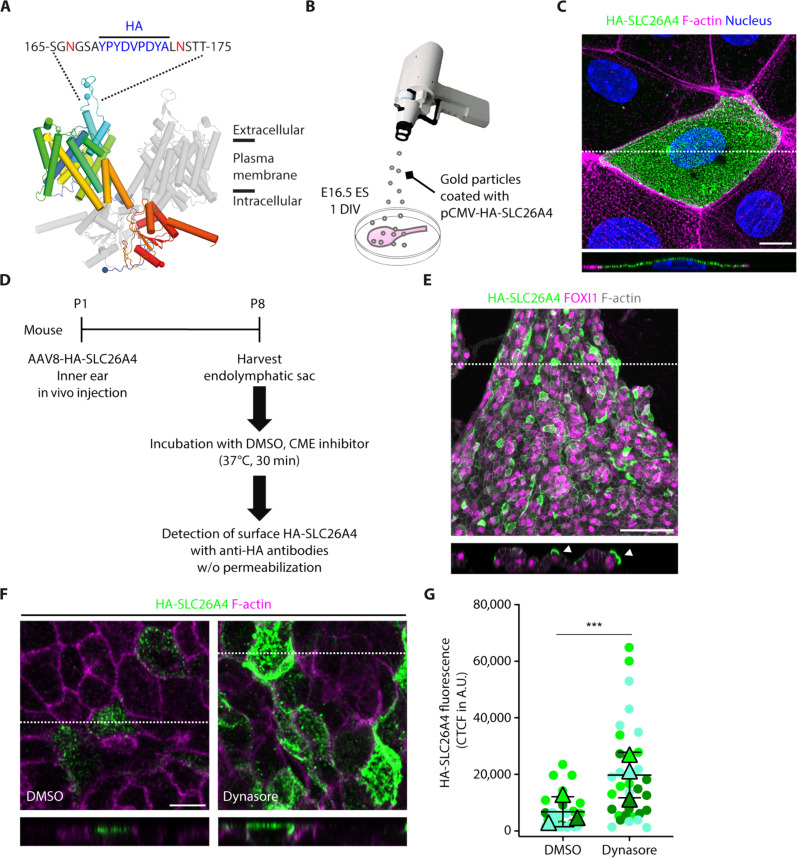
Increased surface localization of AAV8-expressed HA-SLC26A4 in MRCs following pharmacological inhibition of CME. (**A**) HA tag (YPYDVPDYA) was inserted into the longest extracellular loop of SLC26A4, between A170 and L171. (**B**) Schematic of Helios Gene Gun–mediated delivery of plasmid-coated gold particles allowing the expression of HA-SLC26A4 in cells of the E16.5 endolymphatic sac (ES) placed 1 day in culture (DIV). (**C**) Representative image of a transfected ES cell 1 day after plasmid delivery. Anti-HA antibodies were used under nonpermeabilizing conditions to only detect HA-SLC26A4 present at the plasma membrane. HA labeling was concentrated at the apical surface of the cell. (**D**) Experimental approach designed to allow the expression of HA-SLC26A4 in ES in vivo and monitor the changes of its abundance at the plasma membrane following pharmacological inhibition of CME in ex vivo preparations. (**E**) Maximum intensity projection image of ES dissected from mouse inner ear 7 days after injection with AAV8-HA-SLC26A4. HA-SLC26A4 was detected in several cells including some positive for FOXI1, an MRC marker. (**F**) Maximum intensity projection images of DMSO-treated (left, control) and dynasore-treated (right) ESs. HA-SLC26A4 (green) was labeled under nonpermeabilizing conditions with anti-HA antibodies and secondary antibodies coupled to Alexa Fluor 568 (shown here in green). Lower part of each panel: XZ cross section at the level of the dotted line. (**G**) The CTCF was used as a measure of fluorescence associated with the presence of HA-SLC26A4 at the plasma membrane in cells treated with DMSO alone (27 cells) or with dynasore (31 cells) for 30 min. Each dot corresponds to the CTCF of one cell and was color coded by biological replicate. Triangles show the mean value per biological replicate. The mean ± SD of these means is shown. *t* test was used to compare results under the two experimental conditions. The CTCF value for each cell was considered as an independent measurement. A similar result was obtained using Mann-Whitney non-parametric test. ***P < 0.001. Scale bars, 10 μm (C), 50 μm (E), and 5 μm (F).

To test in live tissue if CME is indeed involved in the regulation of SLC26A4 abundance at the plasma membrane of MRCs, we aimed to quantify the changes of SLC26A4 abundance at the plasma membrane induced by pharmacological inhibition of CME. Because none of the antibodies directed against the extracellular regions of SLC26A4 produced a specific signal in MRCs, a hemagglutinin (HA) epitope tag was introduced into the second and longest extracellular loop of SLC26A4, between residues A170 and L171 (Fig. 8A) to monitor its presence at the plasma membrane. If SLC26A4 cell trafficking is not altered by this tag, then anti-HA antibodies used under nonpermeabilizing conditions will allow for the selective detection of HA-SLC26A4 at the cell surface. Using Helios Gene Gun–mediated transfection in endolymphatic sac primary cultures (Fig. 8B), we confirmed that HA-SLC26A4 was expressed and detectable at the plasma membrane. Its enrichment in the apical membrane of the cells, with little to no tagged protein detected at the base of the cells (Fig. 8C), was similar to that observed for endogenous untagged SLC26A4 (Fig. 3D), indicating that the HA tag does not impair HA-SLC26A4 folding or membrane trafficking.

A Helios Gene Gun approach only allows the transfection of a few cells per preparation. To do quantitative studies, we first used an adeno-associated virus (AAV) approach to deliver HA-SLC26A4 to cells of the endolymphatic sac (*36*). AAV8-HA-SLC26A4 was injected into the inner ears of *Tg(ATP6V1B1-EGFP)/+* mice at P1 via the posterior semicircular canal approach (*36*). Seven days later, endolymphatic sacs were microdissected (Fig. 8, D and E). HA-SLC26A4 expressed through AAV8 transduction was found concentrated at the apical membrane of some MRCs identified by their FOXI1 immunoreactivity, a nuclear marker of MRCs (Fig. 8E). Dynasore, a cell-permeable noncompetitive inhibitor of dynamin-1 and dynamin-2 guanosine triphosphatase (GTPase) activity, was used to inhibit CME (*37*). It has been used extensively in internalization assays of a variety of transmembrane cargo proteins in different cell types to block the dynamin-dependent pathway (*37*–*41*). Dynasore (80 μM) applied for 15 to 30 min has been shown to block 90% or more of the activity of dynamin-dependent pathways, acting in seconds to a few minutes depending on the transmembrane cargo proteins and the cell type studied (*37*–*41*). In three sets of experiments, MRCs treated with 80 μM dynasore resuspended in dimethyl sulfoxide (DMSO) (0.8%, v/v) for 30 min at 37°C showed a significant 2.9-fold increase in HA-tagged SLC26A4 present at the plasma membrane [corrected total cell fluorescence (CTCF) = 19,594 ± 17,390 arbitrary units (A.U.), 31 cells], as compared to the amount detected in MRCs treated with DMSO alone (6543 ± 6200 A.U., 27 cells, control) (Fig. 8, F and G).

### The abundance of endogenous HA-SLC26A4 at the plasma membrane in MRCs is also increased by pharmacological inhibition of CME

**Fig. 9.**
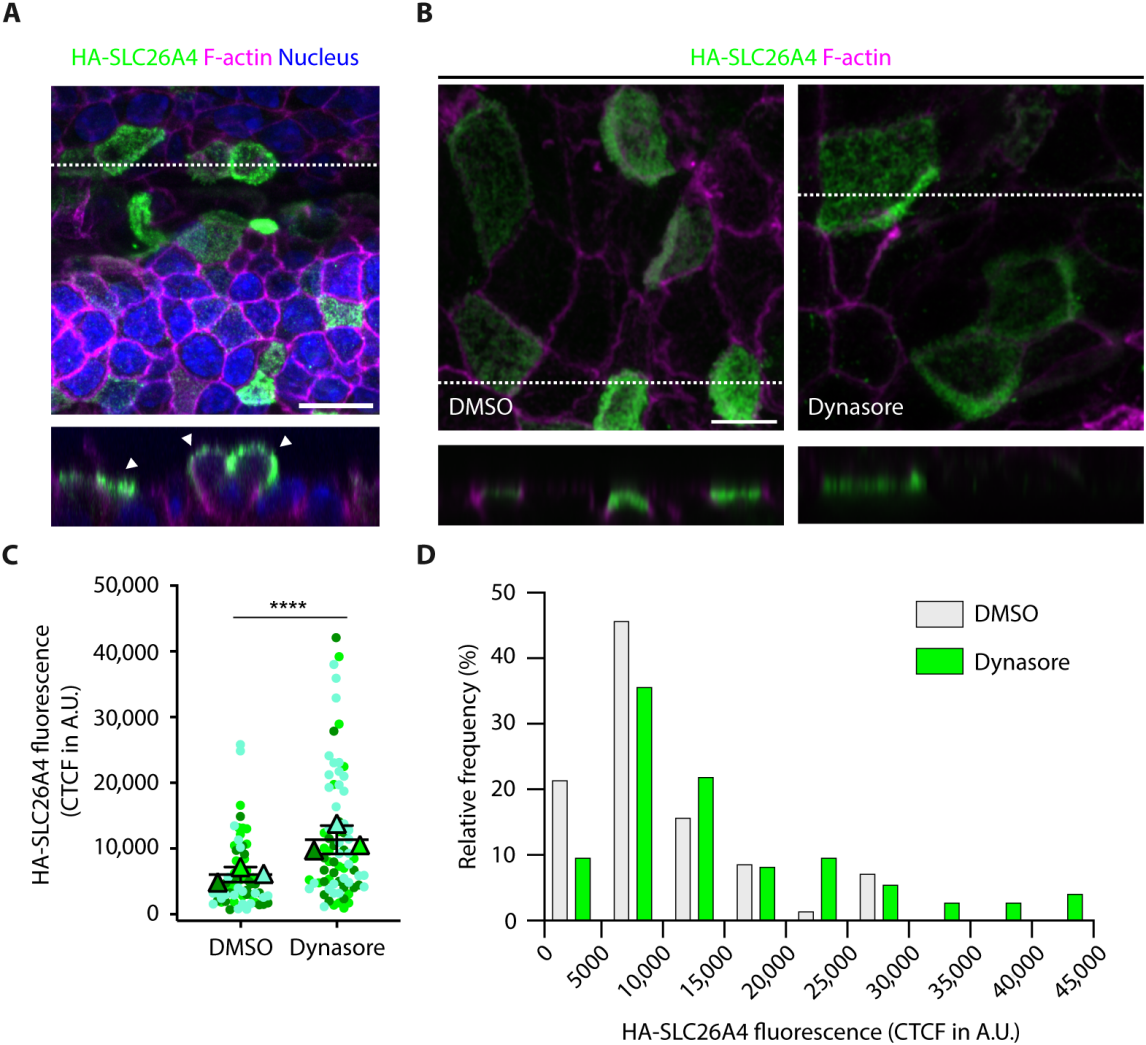
Increased presence of endogenously expressed HA-SLC26A4 at the plasma membrane of the MRCs following pharmacological inhibition of CME. (**A**) Maximum intensity projection images of whole-mount P0 *Slc26a4*^*HA*/+^ mouse endolymphatic sac labeled with anti-HA antibody (green), phalloidin (F-actin, magenta), and Hoechst 33342 (blue) under nonpermeabilizing conditions. Similar to the labeling for the endogenous protein, HA-SLC26A4 labeling is concentrated at the apical membrane of the cells (arrowheads). (**B**) Maximum intensity projection images of P0 *Slc26a4*^*HA*/+^ mouse endolymphatic sacs treated with DMSO alone (left, control) or with dynasore (right). HA-SLC26A4 (green) and F-actin (magenta) were labeled under nonpermeabilizing conditions. Reconstructed XZ cross sections at the level of the white dotted line are shown in the lower panels [(A) and (B)]. (**C** and **D**) Comparison of the results obtained in cells treated with DMSO alone (70 cells, six preparations) or with dynasore (73 cells, seven preparations) for 30 min. The CTCF was used as a measure of the fluorescence associated with the presence of HA-SLC26A4 at the plasma membrane. (C) Each dot corresponds to the CTCF of one cell and was color coded by biological replicate. Triangles show the mean value per biological replicate. The mean ± SD of these means is shown. *t* test was used to compare results under the two experimental conditions. The value for each cell was considered as an independent measurement. A similar result was obtained using Mann-Whitney non-parametric test. (D) Distribution of the cells treated with DMSO alone or with dynasore as a function of their HA-SLC26A4 fluorescence. For these histograms, the bin width was chosen as 5000 A.U. and lower limits are inclusive. ****P < 0.0001. Scale bars, 10 μm (A) and 5 μm (B).

To study the regulation of SLC26A4 localization under endogenous conditions, we generated *Slc26a4*^*HA*/+^ knock-in mice by inserting in-frame the coding sequence of an HA tag in exon 5 of the endogenous *Slc26a4* gene (fig. S8, A and B). In *Slc26a4*^*HA*/+^ mice, surface localization of HA-SLC26A4 was detected in MRCs using anti-HA antibodies under nonpermeabilizing conditions (Fig. 9A). Under permeabilizing conditions, a low level of HA-SLC26A4 immunolabeling was also detectable throughout the cytoplasm using anti-HA antibodies (fig. S9). This is not usually seen as clearly with our anti-SLC26A4 PB826 antibodies. This could be due to a difference in epitope accessibility. No obvious accumulation of protein in the endoplasmic reticulum was detected as expected from a misfolded protein. Arguing in favor of HA-SLC26A4 having WT functionality, mice in which the only source of SLC26A4 was heterozygous expression of HA-SLC26A4 (*Slc26a4*^*HA*/−^ mice) did not show an enlarged endolymphatic sac at an early postnatal age (fig. S10A), a common phenotype seen in mice lacking SLC26A4 expression or function. Moreover, these mice had similar auditory brainstem response (ABR) thresholds as their WT littermates at P30 (fig. S10B). Using classical internalization assays and HA antibodies (*42*), we were able to show the temperature-dependent internalization of endogenously expressed HA-SLC26A4 present at the plasma membrane of MRCs in freshly dissected endolymphatic sacs (fig. S11). Antibodies directed against HA-SLC26A4 were detected in vesicle-like structures in the cytoplasm of MRCs after a 30-min incubation at 37°C. A similar labeling was not detected when the cells were kept for the same time at 4°C, a temperature that blocks endocytosis (fig. S11). This suggests that the signal detected in the cytoplasm corresponds to HA-SLC26A4 internalized by endocytosis.

To further explore the changes of SLC26A4 abundance at the plasma membrane following treatment with dynasore, when SLC26A4 is endogenously expressed, the endolymphatic sacs of *Slc26a4^HA/+^* mice were dissected at P0 to P1, an age when SLC26A4 presence and function are necessary for normal inner ear formation (*10*). MRCs showed a significant increase in fluorescence associated with HA-SLC26A4 presence at the plasma membrane, when treated with dynasore (CTCF = 11,772 ± 9918 A.U., 73 cells, seven preparations) as compared to DMSO alone (7212 ± 6711 A.U., 70 cells, six preparations) (Fig. 9, B to D). These results suggest that SLC26A4 abundance at the plasma membrane in MRCs is regulated, at least in part, by CME.

## DISCUSSION

Worldwide, pathogenic variants in *SLC26A4* are one of the most frequent causes of childhood HL. Patients with HL linked to *SCL26A4* are often born with some level of hearing, which fluctuates over time before decreasing and leading to profound HL. This differs from most other forms of early childhood HL, which are congenital and lack this potential postnatal window of opportunity for therapeutic intervention to stop HL progression. More than 450 pathogenic variants of human *SLC26A4* have been reported, including missense variants leading to mutated SLC26A4 which reach the plasma membrane but are hypofunctional anion exchangers (*43*–*45*). In addition, in about 50% of patients of Caucasian ancestry, HL associated with *SLC26A4* is linked to a specific haplotype (CEVA) (*46*), thought to be associated with a decreased expression of SLC26A4. Here, we aimed at identifying partners of SLC26A4 that could contribute to its regulation. We identified the μ2 subunit of the endocytic adaptor AP-2 as a partner of SLC26A4 using a Y2H screen and further validated this interaction using both Y2H assays in yeast and NanoSPD assays in mammalian cells.

AP-2 is the most abundant endocytic adaptor involved in cargo recognition and clathrin recruitment during endocytosis. Using immunohistochemistry followed by confocal microscopy and immunogold TEM, we showed that AP-2 and SLC26A4 are enriched and partially colocalized in the apical membrane of the MRCs in the mouse endolymphatic sac. SLC26A4 was detected along the microvilli, which cover the apical surface of the MRCs and protrude in the lumen of the endolymphatic sac, but it was also found associated with the CCPs present at their base, where AP-2 is present. This association and the interaction of SLC26A4 with the AP-2 complex linking it to the clathrin scaffold suggested that SLC26A4 turnover and abundance at the plasma membrane of MRCs in the endolymphatic sac could be regulated, at least in part, by CME. Indeed, pharmacological inhibition of the activity of dynamin in the endolymphatic sac led to an increased abundance of HA-SLC26A4 at the plasma membrane of MRCs, bringing further evidence of the relevance of the SLC26A4-μ2 interaction and CME in the regulation of SLC26A4 in the cells where its presence and function are essential for normal inner ear formation and hearing.

Previous studies demonstrated the role of AP-2 in the control of endocytosis of the epidermal growth factor receptor, the trans-Golgi network protein TGN38, the IRS1, and other cargos having YXXΦ motifs in their cytoplasmic tails that interact with a well-defined binding pocket within μ2 (*19*, *29*). We identified four such tyrosine-based motifs in SLC26A4-Ct, two of which are conserved. Our structural analysis of SLC26A4 showed that the motif 536-YKNL in mouse SLC26A4 (536-YKNI in human) was the only motif in extended conformation and with residues available to form an interaction. We confirmed experimentally that mouse SLC26A4 Y536 and L539 were necessary for the interaction of SLC26A4-Ct with μ2. The tyrosine binding motif 536-YKNL, conserved between SLC26A4 orthologs among several mammals and in chicken, turtle, *Xenopus*, and fish (Fig. 5A), is also relatively conserved in the amino acid sequence of the other SLC26 family members, suggesting that their plasma membrane turnover could also be regulated by CME (fig. S12).

Tyrosine-based motifs are known to bind a pocket in μ2 (*19*) that can be divided into two local pockets that respectively bind the tyrosine and the hydrophobic residues of the motif. In our structural models of the SLC26A4-μ2 complex Y536 of both human and mouse, SLC26A4 interacts with the more hydrophilic local pocket in μ2 created by F174, W421, D176, K203, and R423 (Fig. 6D). The aromatic ring of the tyrosine establishes hydrophobic interactions with F174, W421, and the aliphatic chain of R423 and a cation-π interaction with the guanidino group of R423, while its hydroxyl group is involved in a network of hydrogen bonds with D176 and K203. In our experiments, substitution of the residues within the binding pocket of μ2 showed that F174, D176, and W421 are involved in the interaction with SLC26A4 (Fig. 6). Notably, the substitution D176A in μ2 was sufficient to abolish the interaction with SLC26A4-Ct in NanoSPD assays, whereas the additional substitution of F174S or W421S was necessary to see the same effect in Y2H assays. This difference could be due to the different sensitivity of each assay dependent on the molecular mechanisms they are based on.

Few partners of SLC26A4 have been reported. Recently, the interaction of SLC26A4 with the IQ-motif containing GTPase activating protein 1 (IQGAP1) was identified by the Y2H screen of a mouse kidney cDNA library using human SLC26A4 C terminal as the bait (*47*). IQGAP1 colocalizes with SLC26A4 on the apical membrane of type B intercalated cells in the kidney cortical collecting duct, indicating a possible role of IQGAP1 in SLC26A4 trafficking and activity. We also identified 24 clones of IQGAP1 in our Y2H screen, including at least 8 independent clones. Further experiments are needed to test if IQGAP1 contributes to the regulation of SLC26A4 activity in the inner ear and if SLC26A4 abundance at the apical membrane in type B and non-A–non-B intercalated cells of the kidney (*23*) is also regulated by CME.

Previous works had studied protein localization, functional activity, and regulation of SLC26A4 and its variants associated with HL, most often including a C-terminal EGFP tag or analog to follow its localization, and used heterologous cells such as human embryonic kidney (HEK) 293, HeLa, COS-7, or oocytes (*43*–*45*, *48*). Here, we report an alternative approach to tag SLC26A4 without interfering with its N-terminal or C-terminal domains and their potential interactions. We also describe several new methods to study the localization and regulation of SLC26A4 in a more physiological context. In particular, we used primary cultures of the mouse endolymphatic sac, Gene Gun transfections, and in vivo AAV8 transduction to express HA-SLC26A4 to study SLC26A4 regulation in MRCs, which are highly differentiated and polarized cells. The mouse model we developed, expressing endogenously HA-SLC26A4, is a promising tool to study SLC26A4 regulation in the inner ear, thyroid, and kidney. SLC26A4 is important for thyroid function (*49*). It also affects blood pressure (*25*, *50*).

Our results suggest that specific disruption of the ability of SLC26A4 to interact with μ2 could be a target for a therapeutic approach with small molecules to increase SLC26A4 abundance at the cell surface and its function in patients with HL associated with pathogenic missense variants of SLC26A4 that are less abundant at the plasma membrane or able to reach the plasma membrane normally but are hypofunctional. The increased presence of HA-SLC26A4 at the plasma membrane of MRCs following CME inhibition offers a proof of principle to this approach. As CME is essential for cell function, this therapeutic approach must be specific for SLC26A4 without altering the ability of μ2 to bind to other cargos it regulates. It should also not interfere with the ability of other SLC26 family members to bind to μ2. Identification of residues in both SLC26A4 and μ2 that are essential for this interaction to occur and the establishment of a docking model are the first steps to designing small molecules with such specificity. For this purpose, we performed protein-protein docking simulations to obtain a structural model of SLC26A4 bound to μ2, providing atomic-level information on the SLC26A4-μ2 interaction both in mouse and human.

Our study and the therapeutic approach we are suggesting have some limitations. Here, we identified an increase of 63% of SLC26A4 present at the plasma membrane following application of dynasore for 30 min. How much we can slow down the turnover of SLC26A4 by blocking its interaction with μ2 in a continuous manner will need to be determined. Other modes of endocytosis could also contribute to the turnover of SLC26A4 at the plasma membrane, and the ability of these compensatory mechanisms to interfere with decreasing the turnover of SLC26A4 at the plasma membrane should be determined. Disruption of the ability of 536-YKNL to interact with μ2 could also affect the sorting of SLC26A4 between compartments. Further work is needed to identify if 536-YKNL is recognized by other μ subunits, which recognize YXXØ tyrosine-based motifs and are associated with the AP-1, AP-3, or AP-4 complexes that mediate the sorting of transmembrane cargo proteins in post-Golgi compartments and endosomal/lysosomal pathways (*51*–*53*). Although the approach we are proposing has the advantage of not being dependent on the hypomorph variant of *SLC26A4*, the amount of Cl^−^/HCO_3_^−^ anion transport that can be rescued by this approach and if it is sufficient to rescue hearing will depend on the level of remaining activity of the mutated SLC26A4 present in each patient. In patients with HL, local inner ear delivery through a micropump or gel of these small molecules may be preferable to systemic administration of a drug increasing the SLC26A4 half-life at the plasma membrane to avoid regulatory changes of SLC26A4 in the kidney, in particular.

Our results highlight the role of SLC26A4-μ2 interaction and CME in the regulation of SLC26A4 abundance at the plasma membrane of the MRCs in the developing endolymphatic sac. They also identify the potential of interfering with the SLC26A4-μ2 interaction for therapeutic purposes in the inner ear to increase hypofunctional SLC26A4 cell surface abundance and function in patients with such pathogenic variants associated with one of the most frequent forms of HL worldwide.

## MATERIALS AND METHODS

### Study design

We hypothesized that, by studying SLC26A4 interactome, we could identify pathways involved in SLC26A4 regulation. Using Y2H, we identified several independent clones of μ2 subunit of the AP-2 complex, pointing at μ2 as a possible candidate to interact with the C-terminal region of SLC26A4 protein. To test if these two proteins are present in the same cells, at the same time, we first studied single-cell RNA sequencing (scRNA-seq) data that showed that their mRNAs were present in MRCs at the same time, before studying the localization of the two proteins in the developing mouse endolymphatic sac by immunohistochemistry followed by confocal imaging and immunogold EM. Tissues from both male and female mice were studied. SLC26A4 was found not only along the microvilli of the MRCs but also associated with CCPs, where μ2 is present. The interaction between cargo proteins and μ2 is known to be weak and transient, which is one of the reasons why we did not attempt to do coimmunoprecipitation of the endogenous proteins from the endolymphatic sac. The other reason is the small amount of inner ear tissue available per mouse, with each endolymphatic sac containing about 3000 MRCs. Rather, pairwise assays in yeast and NanoSPD assays in HeLa cells were used in parallel to characterize the interaction of SLC26A4 and μ2. Experiments were conducted multiple times and quantified blind to the transfected plasmids. The number of cells and filopodia quantified were defined according to the article describing this approach (*26*). The cryo-EM structures of SLC26A4 (*7*), in combination with our experimental data, allowed the modeling of the mouse and human SLC26A4-μ2 complexes at the atomic level. Lastly, to study the relevance of this interaction in live cells in the inner ear, we aimed at measuring the changes of SLC26A4 abundance at the plasma membrane in MRCs in response to pharmacological inhibition of CME. To quantify the changes of SLC26A4 abundance at the plasma membrane, we developed a reporter protein, SLC26A4, with an extracellular HA tag. To test potential misfolding and mislocalization due to this tag, primary cultures of mouse endolymphatic sacs were used along with a Gene Gun approach to express HA-SLC26A4. The presence of the HA tag did not interfere with SLC26A4 trafficking to the apical membrane of MRCs. We then quantified the effect of blocking CME on HA-SLC26A4 plasma membrane abundance using a short application of the dynamin inhibitor, dynasore, to limit potential indirect effects of blocking CME. To study SLC26A4 regulation in the cells where SLC26A4 is essential for hearing and is expressed under the influence of the same physiological environment in the very particular epithelium of the endolymphatic sac, experiments were first conducted in acutely dissected endolymphatic sacs where HA-SLC26A4 had been expressed using AAV8 delivery in the inner ear. To bypass the limitations of an overexpressing system, we also used CRISPR-Cas9 to insert the coding sequence of the HA tag in the endogenous *Slc26a4* gene and expressed the reporter protein at endogenous levels*.* To test a potential deleterious effect of the HA tag on SLC26A4 function, we studied endolymphatic sac morphology and hearing in these mice. Both were similar to those of their WT littermates, even when the only source of SLC26A4 had the HA tag (*Slc26a4^HA/−^* mice). In MRCs either overexpressing HA-SLC26A4 or expressing it endogenously, a significant increase in HA-SLC26A4 abundance at the cell surface was detected in the presence of dynasore as compared to controls in multiple biological replicates, providing evidence of the relevance of the SLC26A4-μ2 interaction in live cells and of the role of μ2 and CME in the regulation of the abundance of SLC26A4 at the plasma membrane of the MRCs. In all experiments, inhibitor or control treatment was allocated randomly to the preparations. No data were excluded.

Detailed materials and methods are provided in the Supplementary Materials.

### Ethics declaration and animal information

All animal experiments and procedures were performed according to protocol 1264 approved by the Animal Care and Use Committee (ACUC) of the National Institute of Neurological Disorders and Stroke and National Institute on Deafness and Other Communication Disorders at the National Institutes of Health (NIH). C57BL/6J mice (no. 000664, RRID: IMSR_JAX:000664) and B6.CBA-*Tg(ATP6V1B1-EGFP)^1Rnel/Mmjax^* mice (no. 010704, RRID:MMRRC_032047-JAX) are available through the Jackson Laboratory.

### Y2H screen and pairwise Y2H assays

ULTImate Y2H analysis was performed by Hybrigenics Services (https://hybrigenics-services.com/). The cDNA of mouse *Slc26a4* (NM_011867.3) was obtained by reverse transcription polymerase chain reaction from microdissected E16.5 C57BL/6J mouse endolymphatic sac mRNA.

### NanoSPD assays

The cDNAs corresponding to mouse *Ap2m1* FL, MID, and Ct were subcloned using the cDNA of HA-tagged *Ap2m1* in pCI-Neo as the template (*54*). NanoSPD assays were performed as described (*26*). To test each interaction, two to three independent experiments were conducted with a maximum of 10 filopodia counted per cell and at least 24 cells studied per experimental condition.

### Endolymphatic sac immunohistochemistry

Immunohistochemistry was performed as described (*55*) with minor variations. E16.5 mouse inner ears were labeled with rabbit anti-SLC26A4 (PB826, RRID: AB_2713943) and mouse anti–α-adaptin (ab2730, Abcam). Hoechst 33342 (H3570, Invitrogen) was used to label cell nuclei. Whole-mount specimens were imaged using a Zeiss LSM 880 confocal microscope. Labeling results from tissues from three males and three females were compared to test for sex differences.

### TEM and immunogold EM

TEM studies of ultrastructure of four endolymphatic sacs from P0 and P5 males and females were performed as described previously and examined in a JEOL JEM-1400 TEM or a JEOL JEM-2100 TEM. Post-embedding immunogold studies were performed on four endolymphatic sacs (P0; two males and two females) using rabbit anti-SLC26A4 antibody (PB826). Control experiments conducted in the absence of primary antibodies only showed rare gold particles (fig. S1).

### Sequence comparison

Sequences obtained from UniProt were analyzed in Jalview. Alignment was performed using ClustalW (*56*). In Fig. 5A, the sequence identifiers for SLC26A4 orthologs are Q9R155, O43511, A0A8C0SH80, G3T5S4, F7F6U5, A0A452G5H8, A0A8V0Y9J3, K7G6W6, Q6DCJ6, and C8XTB7. Residues were colored according to their physicochemical properties: hydrophobic (white), aromatic (orange), positively charged (blue), polar (green), and glycine (pink).

### Loop remodeling in the SLC26A4 dimer

Loop remodeling technique was applied to the mouse SLC26A4 structure (PDB ID: 7wk1) (*7*) to identify the most probable configuration of the 536-YKNL tyrosine binding motif and neighboring residues (Y-loop). Three different loop lengths were used, defined as loop 1 (533 to 541) that contained all the residues in the Y-loop and two shorter versions, loop 2 (534 to 541) and loop 3 (535 to 541). For each loop, a pool of 10,000 models was generated with the MODELLER software package. The resulting models in each pool were structurally superimposed onto their transmembrane domain and then clustered using the simple exclusive clustering algorithm from Daura *et al.* implemented in the Gromacs software (*57*, *58*) using 1.5, 2, 2.5, 3, 3.5, and 4 Å as root mean square deviation (RMSD) cutoffs. The RMSD cutoff selected for each run was the lowest with the highest cluster occupancy (RMSD 2.5 Å for loops 1, 2, and 3). Last, the representative of the most populated cluster in loop 1 (mSLC26A4 loop 1) was used as the input in the protein-protein docking runs, while those of loops 2 and 3 were discarded due to lower solvent accessibility of the tyrosine-based motif residues in these models.

### Modeling of the SLC26A4-μ2 interaction

The complex mSLC26A4-loop1-μ2 was obtained with protein-protein docking simulations of the mSLC26A4-loop 1 model and μ2 protein structure obtained from the x-ray structure of human μ2 (residues 161 to 435) [PDB ID: 6bnt (*29*)] using the HADDOCK server. Two sets of docking simulations (run1, run2) (named A1-4 and B1-4, respectively) were designed using different conditions. In runs A1-4, the initial relative orientation of SLC26A4 and μ2 was randomized as per the standard procedure of the HADDOCK docking protocol, while in runs B1-4, the initial relative orientation was that obtained for the best initial mSLC26A4-μ2 complex selected from run1. Last, backbone flexibility for the residues remodeled in the loop-remodeling step (533 to 541 in loop 1) was introduced in runs A3-4 and B3-4. For each run, a set of 2000 models of the complex was generated where only the 500 highest scored models were solvated and only the subsequently 200 highest scored models were clustered. In addition, distance restraints extracted from the PDB structure of human μ2 bound to a tyrosine peptide (PDB ID: 6bnt) were used (table S2), which in runs A2, A4, B2, and B4 had ±0.3 Å of deviation applied. Lastly, residues in the μ2 structure that participate in the binding of the tyrosine peptide (174, 176, 203, 401, 420, 422, and 423) together with Y536 and L539 of SLC26A4 were defined as active residues while passive residues in both proteins were defined by HADDOCK using default parameters (fig. S3).

The model with the best energy score within those representing the most populated clusters of A1-4 docking simulations was selected as the input for B1-4 protein-protein runs. The resulting best scored model within those belonging to the most populated clusters of each run (B1-4) was selected as the final mSLC26A4-μ2 model. This final model was then used as a template to model the human SLC26A4-μ2 complex using the MODELLER software. The final human SLC26A4-μ2 complex was the one with the best MolPDB score among the 2000 models that were generated using the alignment in fig. S4.

### Helios Gene Gun expression of HA-SLC26A4 in the endolymphatic sac explant culture

Endolymphatic sacs from E16.5 C57BL/6J mice (males and females), were microdissected in ice-cold Hanks’ balanced salt solution (HBSS; Gibco) then transferred to permeable supports or glass-bottom dishes (no. 1.5, MatTek) and grown in Dulbecco’s modified Eagle’s medium (DMEM)/F12 (no. 11039021, Gibco) supplemented with 10% (v/v) heat-inactivated fetal bovine serum (Invitrogen) and GlutaMAX at 37°C and 10% CO_2_. Results presented here were from tissues incubated on glass-bottom dishes. The HA tag (YPYDVPDYA) was inserted between residues A170 and L171 predicted to be located into the second and longest extracellular loop of mouse SLC26A4 using an In-Fusion cloning kit (Takara Bio) and primers 5′-GTATGATGTTCCGGATTATGCATTGAACTCGACCACGTTAGACA-3′ and 5′-TCCGGAACATCATACGGATATGCACTTCCGTTACCGCTG-3′. HA-SLC26A4 was then expressed in E16.5 endolymphatic sac explant cultures by Helios Gene Gun–mediated transfection as previously described (*59*). Briefly, pCMV vector containing HA-*Slc26a4* cDNA-coated gold particles were applied to the explant cultures using the Helios Gene Gun delivery system (Bio-Rad Laboratories). Twenty-four hours after transfection, cultures were fixed in 4% paraformaldehyde (PFA) and labeled with anti-HA antibodies (1:200, Cell Signaling Technology) under nonpermeabilizing conditions. Alexa Fluor 568–phalloidin and Hoechst 33342 were used to stain the F-actin and nucleus, respectively.

### In vivo AAV8-driven expression of HA-SLC26A4 in the endolymphatic sac

AAV8 (serotype 2/8) allowing the expression of HA-tagged *Slc26a4* cDNA was produced by Vector Biolabs (Malvern, PA, United States). The vector packaged in AAV8 capsid contained a cytomegalovirus (CMV) promoter and bovine growth hormone polyadenylation signal. To express HA-SLC26A4 in the endolymphatic sac in vivo, 2 μl of AAV8 pCMV-HA-*Slc26a4* at a final titer of 4.2 × 10^12^ genome copies/ml were injected in the inner ear of B6.CBA-*Tg(ATP6V1B1-EGFP)^1Rnel/Mmjax/+^* neonate mice (P1) via the posterior semicircular canal as described (*36*). The expression of EGFP in the endolymphatic sac facilitates the dissection of this monolayer epithelium at postnatal ages. Briefly, pups were anesthetized by hypothermia and surgery was performed in the left ear of each animal. Once a post-auricular incision was made and the posterior semicircular canal was exposed, AAV8 was injected using a Nanoliter Microinjection System (Nanoliter2000, 2020; World Precision Instruments) and a glass micropipette with a flow rate of 40 nl/s. The incision was closed with 5-0 Vicryl sutures and tissue glue. Injected pups recovered on the heating pad before being reintroduced to their mother.

### Generation using CRISPR-Cas9 editing of a knock-in mouse expressing endogenously HA-tagged SLC26A4

One copy of the 27-bp HA tag coding sequence was inserted in between chr12:31,547,936-31,547,935 (GRC m38/mm10) in exon 5 of mouse *Slc26a4* by CRISPR-mediated homologous recombination with a single-stranded oligonucleotide as the recombination template (see the Supplementary Materials and fig. S8, A and B). The line *B6.Cg-Slc26a4^em1Iroux/+^* (*Slc26a4^HA/+^*) was obtained.

### Pharmacology on freshly dissected endolymphatic sacs and HA-SLC26A4 surface quantification

To quantify potential changes in the surface abundance of HA-SLC26A4 induced by inhibition of CME, endolymphatic sacs were collected either 7 days after AAV8-HA-SLC26A4 inner ear microinjection or at P0 to P1 when studying *Slc26a4^HA/+^* mice. Microdissected endolymphatic sacs harvested and kept in ice-cold HBSS were incubated for 30 min at 37°C either with DMSO (0.8%, v/v) alone or with dynasore hydrate (80 μM; D7693, Sigma-Aldrich) in a serum-free DMEM/F12 growth medium (*37*). Preparations were randomly chosen to receive one treatment or the other. Explants were then fixed in 4% PFA and incubated with anti-HA antibodies (1:200; 3724S, Cell Signaling Technology) without permeabilization. For nuclear staining, Hoechst 33342 (1:500; H3570, Invitrogen) was used. For AAV8-injected endolymphatic sacs, anti-FOXI1 antibodies (ab20454, Abcam) were used to label MRCs after permeabilization with 0.2% Triton X-100 in blocking solution (10% normal donkey serum in phosphate-buffered saline) to allow for MRC identification. HA fluorescence was imaged by Airyscan confocal microscopy (Zeiss LSM 880). Z-stack images were analyzed in ImageJ to quantify the abundance of HA-SLC26A4 at the cell surface. The CTCF was calculated using the ImageJ software. CTCF = Integrated Density − (Area of selected cell × Mean fluorescence of background readings).

### Data presentation and statistics

Quantifications are presented as SuperPlots showing individual measurements as dots obtained for each biological replicate (*N*) in different colors (*60*). For each experimental condition, triangles show the mean value per biological replicate. The mean ± SD of these means is shown when *N* > 2. All statistical testing was performed in Prism (v9.0, GraphPad). Comparisons between two groups were done using Student’s *t* test. For multiple comparisons, one-way or two-way analysis of variance (ANOVA) was used with Tukey’s post hoc tests. In these tests, individual values obtained in each experimental condition were compared. Differences were considered statistically significant for *P* values < 0.05.

## Supplementary Materials

### The PDF file includes:

Supplementary Materials and Methods Figs. S1 to S12 Tables S1 to S4 Legend for data file S1 Legend for structural models of mouse SLC26A4-μ2 Legend for structural models of human SLC26A4-μ2 Legend for file used to obtain the structural models of mouse and human SLC26A4 complexed with AP-2 References

### Other Supplementary Material for this manuscript includes the following:

Data file S1

Structural models of mouse SLC26A4-μ2

Structural models of human SLC26A4-μ2

File used to obtain the structural models of mouse and human SLC26A4 complexed with AP-2
