# Supplementary material for: SLC26A4-AP-2 mu2 interaction regulates SLC26A4 plasma membrane abundance in the endolymphatic sac
Source: Sci Adv. 2024 Oct 9;10(41):eadm8663. doi: 10.1126/sciadv.adm8663 (PMC11638888; doi:10.1126/sciadv.adm8663)
Supplement: Supplementary file 1 — Supplementary Materials and Methods Figs. S1 to S12 Tables S1 to S4 Legend for data file S1 Legend for structural models of mouse SLC26A4-μ2 Legend for structural models of human SLC26A4-μ2 Legend for file used to obtain the structural models of mouse and human SLC26A4 complexed with AP-2 References [file sciadv.adm8663_sm.pdf]

Science Advances
AAAS

Supplementary Materials for

SLC26A4-AP-2 mu2 interaction regulates SLC26A4 plasma membrane abundance in the endolymphatic sac

Hyun Jae Lee *et al.*

Corresponding author: Isabelle Roux, isabelle.roux@nih.gov

Sci. Adv. **10**, eadm8663 (2024)
DOI: 10.1126/sciadv.adm8663

The PDF file includes:

Supplementary Materials and Methods
Figs. S1 to S12
Tables S1 to S4
Legend for data file S1
Legend for structural models of mouse SLC26A4-μ2
Legend for structural models of human SLC26A4-μ2
Legend for file used to obtain the structural models of mouse and human SLC26A4 complexed with AP-2
References

Other Supplementary Material for this manuscript includes the following:

Data file S1
Structural models of mouse SLC26A4-μ2
Structural models of human SLC26A4-μ2
File used to obtain the structural models of mouse and human SLC26A4 complexed with AP-2

Supplementary Materials and Methods

Animals

Mice were housed in PNRC, NINDS/NIDCD Animal Facility at NIH IRP, Bethesda, MD, USA. This facility has received approval from the Office of Laboratory Animal Welfare (OLAW) for their Animal Welfare Assurance (D16-00602 (A4149-01)), a document setting forth the responsibilities and procedures of the Institution regarding the care and use of laboratory animals according to the Public Health Service policy.

It has also received AALAAC International accreditation. Mice were housed in a controlled temperature environment on a 12 h light–dark cycle. Food and water were provided *ad libitum*. E16.5 to P30 mice were used in this study. Both males and females were included. B6.CBA-*Tg(ATP6V1B1-EGFP)*$^{1Rnel/Mmjax}$ mice were previously reported (*61*). The first day after overnight mating was counted as E0.5. The sex of embryos was determined by PCR as published (*62*).

Yeast two-hybrid (Y2H) screen

The coding sequence for the cytosolic carboxy-terminal region of mouse SLC26A4 (NP_035997.1: aa 512-780) was subcloned into pB27 to express it fused to the carboxy-terminal domain of LexA DNA binding domain (LexA-SLC26A4-Ct) and used as bait. A randomly primed adult mouse kidney cDNA prey library (C57BL/6J, age 8-10 weeks) including 13.5 million independent fragments cloned into pP6, was used to identify potential partners of SLC26A4-Ct by Y2H. pB27 and pP6 derive from the original pBTM116 (*63*) and pGADGH (*64*) plasmids, respectively. 90 million interactions were screened using a mating approach with YHGX13 (Y187 ade2-101::loxP-kanMX-loxP, matα) and L40ΔGal4 (mata) yeast strains as previously described (*65*). This corresponds to a coverage of almost seven times the library complexity. The DO-3 selective medium without tryptophan, leucine, and histidine was used to identify an interaction between bait and prey. This led to the growth of 319 clones. The prey fragments of these positive clones were amplified by PCR and sequenced at their 5' and 3' junctions. The resulting sequences were used to identify the corresponding interacting proteins in the GenBank database (NCBI) using a fully automated procedure. A confidence score (PBS, for Predicted Biological Score) was attributed to each interaction as described (*66*). This led to the identification of 21 different potential interacting partners including μ2.

Y2H pairwise assays

To further validate and characterize the protein interactions identified in the Y2H screen, additional Y2H assays were performed by Hybrigenics SA. LexA-SLC26A4-Ct subcloned into pB27 was used as bait. The cDNA corresponding to mouse μ2-MID (NP_033809.1: aa 124 – 435) was cloned into pP6 to expressed μ2 fused to the carboxy-terminal part of Gal4 activation domain (Gal4 activation domain-μ2) and was used as prey. Bait and prey constructs were transformed into yeast haploid cells L40ΔGal4 and YHGX13, respectively. The diploid yeast cells were obtained using a mating protocol with both yeast strains (*65*). These assays are based on the expression of the HIS3 reporter gene and the ability of the yeast to grow without histidine when bait and prey interact. As negative controls, the bait plasmid was tested in the presence of empty prey vector (pP6) and the prey plasmid with the empty bait vector (pB27). The interaction between SMAD and SMURF was used as positive control (*67*). Controls and interactions were tested in the form of streaks of three independent yeast clones for each control and interaction on DO-2 and DO-3 selective media. The DO-2 selective medium lacking tryptophan and leucine was used as a growth

control and to verify the presence of the bait and prey plasmids. The DO-3 selective medium was used to identify interaction between bait and prey. The cDNAs corresponding to mutated SLC26A4-Ct and μ2-MID were cloned into pB27 and pP6 plasmids, respectively.

Nanoscale pulldown assays

Vectors expressing mCherry-tagged myosin-10 heavy meromyosin-like domain ($MYO10^{HMM}$ designated here as MYO10) and EGFP-tagged MYO10 for NanoSPD assays were described (*26*). For these assays, the bait was fused to either mCherry-tagged MYO10 or EGFP-tagged MYO10 available from Addgene (plasmid 87256; https://www.addgene.org) and the prey was fused to either EGFP or mCherry. PCR amplification and subcloning were performed using In-Fusion cloning (Takara Bio, Shiga, Japan). Site-directed mutagenesis was performed using the In-Fusion HD EcoDry cloning (Takara Bio) with the recombinant plasmid of mCherry-MYO10-SLC26A4-Ct^{BAIT} and μ2-MID-$EGFP^{PREY}$ as templates. Plasmid DNAs for transfections were prepared endotoxin-free (NucleoBond Xtra Midi EF; Takara Bio) and the sequence of the inserts of all expression constructs were verified by Sanger sequencing. Briefly, HeLa cells (CCL-2; ATCC, Manassas, VA, USA) were grown in Dulbecco's Modified Eagle Medium (DMEM, #11965092, Gibco) supplemented with 10% (vol/vol) heat-inactivated fetal bovine serum (FBS, #A3840001, Invitrogen) and GlutaMAX (#35050061, Gibco) and incubated at 37°C, 10% CO_2. HeLa cells were transfected with Lipofectamine 3000 (#L3000001, Invitrogen) following the manufacturer's instructions. Transfected cells were incubated for 16–20 h before being seeded on fibronectin-coated (10 μg/ml; Sigma) glass-bottom culture dishes (#1.5, MatTek). Cells were then incubated for 6–8 h before fixation with 4% PFA (Electron Microscopy Sciences) diluted in phosphate-buffered saline (PBS, Invitrogen) for 15 min. Fixed cells were stained with Atto390 phalloidin (# 50556, Sigma) or Alexa Fluor 647 Phalloidin (#A22287, Invitrogen) and imaged using a Zeiss LSM 880 confocal microscope (Carl Zeiss). The confocal images of single cells were imported in FIJI software where they were analyzed blind to the plasmids transfected. The fluorescence intensities of bait and prey at filopodia tips were quantified and further analyzed as described (*26*).

Endolymphatic sac immunohistochemistry

Immunohistochemistry was performed as described (*55*) with minor variations. E16.5 mouse inner ears were harvested and fixed for 1 h at 4°C in 4% paraformaldehyde (PFA), freshly diluted in phosphate-buffered saline (PBS). After fixation, the tissue was washed three times in PBS. The endolymphatic sacs were microdissected, permeabilized for 30 min in PBS with 0.5% Triton X-100 (X100, Sigma) and blocked for 1 h with 5% normal donkey serum (#566460, Sigma) at room temperature (RT). Samples were then incubated with rabbit anti-SLC26A4 (PB826, RRID:AB_2713943, diluted 1:400) and mouse anti-α-adaptin (ab2730, Abcam, Cambridge, UK, diluted 1:200) in PBS with 5% normal donkey serum overnight at 4°C. The specificity of PB826 has been previously shown in the endolymphatic sac tissue using $Slc26a4^{\Delta 8/\Delta 8}$ mouse endolymphatic sac as negative control (*10*). After three washes in PBS, the samples were incubated with Alexa Fluor-conjugated secondary antibodies (1:700, Thermo Fisher Scientific) diluted in PBS for 1 h at RT. Hoechst 33342 (H3570, Invitrogen) was included with the secondary antibodies to label cell nuclei. Microdissected endolymphatic sacs were washed three times in PBS and mounted in ProLong Gold Antifade Mountant (P36934, Invitrogen). Whole-mount specimens were imaged using a Zeiss LSM 880 confocal microscope. Labeling results from tissues from 3 males and 3 females were compared to test for sex differences.

Transmission electron microscopy (TEM)

TEM studies of ultrastructure of 4 endolymphatic sacs from P0 and P5 males and females were performed as described previously (*68, 69*). The tissue including the endolymphatic sac was fixed in 4% PFA plus 2% glutaraldehyde in 0.1 M phosphate buffer (PB), washed in PB, dissected further, and then washed in 0.1 M cacodylate buffer, fixed in 2% glutaraldehyde, washed, and fixed in 1% osmium tetroxide, dehydrated in alcohols with 1% uranyl acetate in the 50% step, transferred to propylene oxide and embedded in Epon. Sixty nanometer (nm) sections were placed on single-slot grids (EMS) and stained with uranyl acetate and lead citrate and examined in a JEOL JEM-1400 TEM or a JEOL JEM-2100 TEM.

Immunogold electron microscopy

Post-embedding immunogold electron microscopy studies were performed on 4 endolymphatic sacs as described (*68, 70*). Tissue with endolymphatic sacs were fixed in 4% PFA with 0.5% glutaraldehyde in PB, washed in PB with 4% glucose, dissected further, and cryoprotected in glycerol/buffer overnight. Tissue was then frozen in a Leica EM CPC, and processed for embedding in Lowicryl (Electron Microscopy Sciences) in a Leica AFS freeze-substitution instrument. Thin sections were treated with 0.1% sodium borohydride plus 50 mM glycine in Tris-buffered saline with 0.1% Triton X-100 (TBST), then with 10% normal goat serum (NGS) in TBST, and subsequently incubated in the presence of rabbit anti-SLC26A4 antibody (PB826; 1/15) in 1% NGS-TBST overnight. The tissue was then washed and incubated in immunogold-conjugated goat anti-rabbit (10 nm) antibodies (1/40; Ted Pella, diluted in 1% NGS-TBST with 0.5% polyethylene glycol (20,000 M.W.)), washed, and stained with uranyl acetate and lead citrate.

Engineering of the mouse line expressing endogenously HA-tagged SLC26A4 through CRISPR-mediated homologous recombination in zygotes of C57BL/6J

One copy of the HA tag coding sequence (27 bp) was inserted in-frame between codons 170, coding for amino-acid Ala, and 171, coding for Leu, in exon 5 of the mouse *Slc26a4* gene by CRISPR-mediated homologous recombination with a single strand oligonucleotide as the recombination template. A pair of guide RNAs (gRNA, for SpCas9, PAM=NGG) flanking the insertion point was selected based on its position to the insertion point and ranking score by the online gRNA selection tool (www.CRISPRscan.org). The gRNA pair was synthesized with T7 *in vitro* transcription as described (*71*) and further tested for its efficiency of *in vitro* cleavage and indel mutagenesis activity in cell culture. For the *in vitro* cleavage assay, genomic PCR product containing the target sites of selected gRNAs was incubated with SpCas9 protein (NEB, New England Biolabs) following the manufacturer's suggested protocol and results of this experiment were analyzed on a 2% agarose gel stained with ethidium bromide. gRNAs were further tested for their efficiency to induce indels at target sites in an immortalized mouse embryonic fibroblast (MEF) cell line engineered to carry a tet-inducible Cas9 expression cassette. Upon confirmation of efficient target-cleavage activity in MEF cells, the selected two gRNAs were mixed with SpCas9 protein (PNA Bio) to form ribonuclear particles (RNPS) by incubating at 37°C for 15 min, and a 200 bp recombination oligonucleotide (100 ng/µl) was added to the preparation. The mixture of RNPs and oligo was microinjected into mouse zygotes as described (*72*). The oligonucleotide was constructed so that the HA tag sequence was flanked by 5' and 3' homology arms of 75 and 90 bp, respectively, to serve as a template of gap repair

by homologous recombination. The two gRNAs used to generate the HA knock-in allele at the endogenous mouse *Slc26a4* gene were the upstream gRNA 5’ CCACTTTCTTGTGCCCAG 3’ and the downstream gRNA 5’ GTGCTTGCAAGCAACACTC 3’. The sequence of the recombination oligonucleotide was 5’ TTAATGGTGGGATCTGTTGTTCTGAGCATGGCTCCAGATGACCACTTTCTTGTGCCa AGtGGTAACGGAAGTGCAtacccatacgatgttccagattacgctTTGAACTCGACCACGTTAGACA CTGGAACCAGAGATGCGGCaCGAGTGTTGCTTGCAAGCACACTCACTCTTCTAGTT GGAATCATACAGgtagtaga 3’.

F0 mosaic founder mice were screened for the presence of the knock-in allele by PCR, TA cloning and sequencing (table S3). One F0 founder (#4664) was found to carry the intended HA knock-in allele. This founder was backcrossed to C57BL/6J mice to obtain germline transmission of the $Slc26a4^{HA}$ allele, and establish the line $B6.Cg\text{-}Slc26a4^{em1Iroux/+}$ ($Slc26a4^{HA/+}$). Primers used for subsequent genotyping are included in table S4.

Characterization of $Slc26a4^{HA}$ allele

To test whether the insertion of the HA tag significantly altered the level of expression of SLC26A4 or its function, $Slc26a4^{HA/-}$ mice were studied. These mice carry a null allele of *Slc26a4* ($Slc26a4^{c.1004insG}$, carrying the variant NM_011867.4:c.1004insG, p.(Phe335Cysfs41)), *in trans* with $Slc26a4^{HA}$, making HA-SLC26A4 the expected only source of the protein.

Endolymphatic sac morphology

Hemi-heads of P0–P1 $Slc26a4^{HA/-}$ pups and $Slc26a4^{+/+}$ control littermates were fixed with 4% PFA for 1 h at 4°C. After washing with ice-cold PBS twice, the endolymphatic sacs were microdissected in ice-cold PBS. The samples were then transferred to glass-bottom dishes (# 1.5, MatTek), and imaged with a stereo microscope (SteREO Discovery V8, Zeiss).

Auditory brainstem responses (ABR)

As C57BL/6J mice carry a recessive variant in *Cdh23* (*Ahl*, NM_023370.3:c.753G>A), making them susceptible to age-related hearing loss (*73*, *74*), $Slc26a4^{HA/+}$ mice were bred with *Cdh23* wild-type ($Cdh23^{753G}$) mice with a C57BL/6J background (B6.CAST-$Cdh23^{Ahl+/Kjn}$, #002756). Auditory brainstem responses (ABR) were measured at P30 in mice heterozygous for the wild-type allele $Cdh23^{Ahl/+}$. $Slc26a4^{HA/+}$ mice were crossed with $Slc26a4^{+/-}$ mice to test for an effect of HA tagging under *Slc26a4* null background.

Mice were anesthetized with ketamine (56 mg/kg body weight) and dexmedetomidine (0.375 mg/kg body weight) via intraperitoneal injection and placed on a heating plate (ATC-2000, World Precision Instruments). Stimulus generation and ABR recordings were completed using Tucker-Davis Technologies hardware (RZ6 Multi I/O Processor; Tucker-Davis Technologies) and software (BioSigRx, v.5.1). ABR thresholds were measured blind to the genotype of the mice, at 8, 16, 32 and 40 kHz using 3-ms, Blackman-gated tone pips presented at 29.9/s with alternating stimulus polarity. 512–1024 responses were averaged at each stimulus level. Thresholds were defined as the lowest stimulus level at which any brainstem response could be reliably detected. This data analysis was performed blind to the genotype of the mice.

HA-SLC26A4 internalization assays

Endolymphatic sacs from E16.5 $Slc26a4^{HA/+}$ mice were micro-dissected in ice-cold HBSS and placed on a glass-bottom dish on ice. The endolymphatic sacs were opened to expose their luminal

side and incubated with anti-HA antibody (1:200; 3724S, Cell Signaling) for 20 min on ice. Antibodies not bound to HA-SLC26A4 present at the cell surface were then removed, and preparations were washed once with ice cold PBS, before being incubated in serum-free medium (DMEM-F12). Endolymphatic sac preparations were then incubated for 30 min either on ice or at 37°C to allow internalization of anti-HA antibodies bound to HA-SLC26A4. The explants were then immediately transferred on ice and fixed for 10 min with ice cold 4% PFA diluted in PBS. Surface expression of HA-SLC26A4 was detected by incubation with Alexa Fluor 568-conjugated anti-rabbit secondary antibodies without permeabilization. After PBS washes, the preparations were permeabilized and blocked with 0.5% Triton X-100, 5% normal donkey serum diluted in PBS for 30 min on ice. Total HA-SLC26A4 was detected by incubation with Alexa Fluor 488-conjugated anti-rabbit secondary antibodies. Fluorescence was analyzed by confocal microscopy (Zeiss LSM 880). HA-SLC26A4 labeling present in the cytoplasm detected in permeabilizing conditions corresponds to HA-SLC26A4 internalized during the 30 min incubation at 37°C.

Supplementary Figures

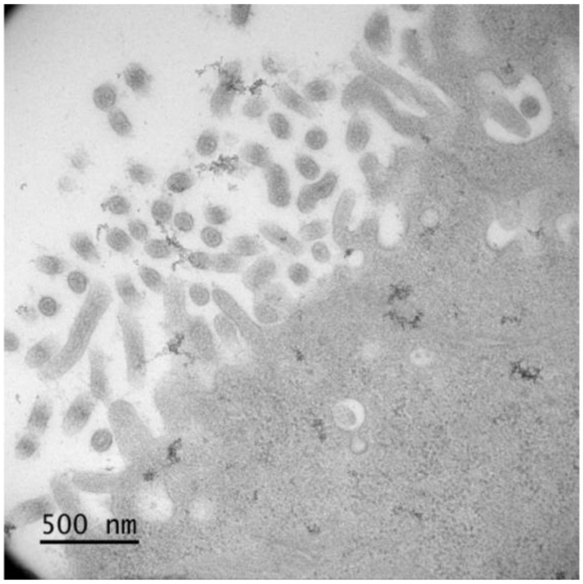

Fig. S1. Representative immunogold-electron microscopy image of endolymphatic sac control experiments.

In these experiments, the sections from 4 animals (2 males and 2 females) were labeled as described for Fig. 4 H-J, but primary antibodies were omitted. The secondary antibodies coupled to 10 nm gold particles did not show significant binding to the tissue section. Scale bar: 500 nm.

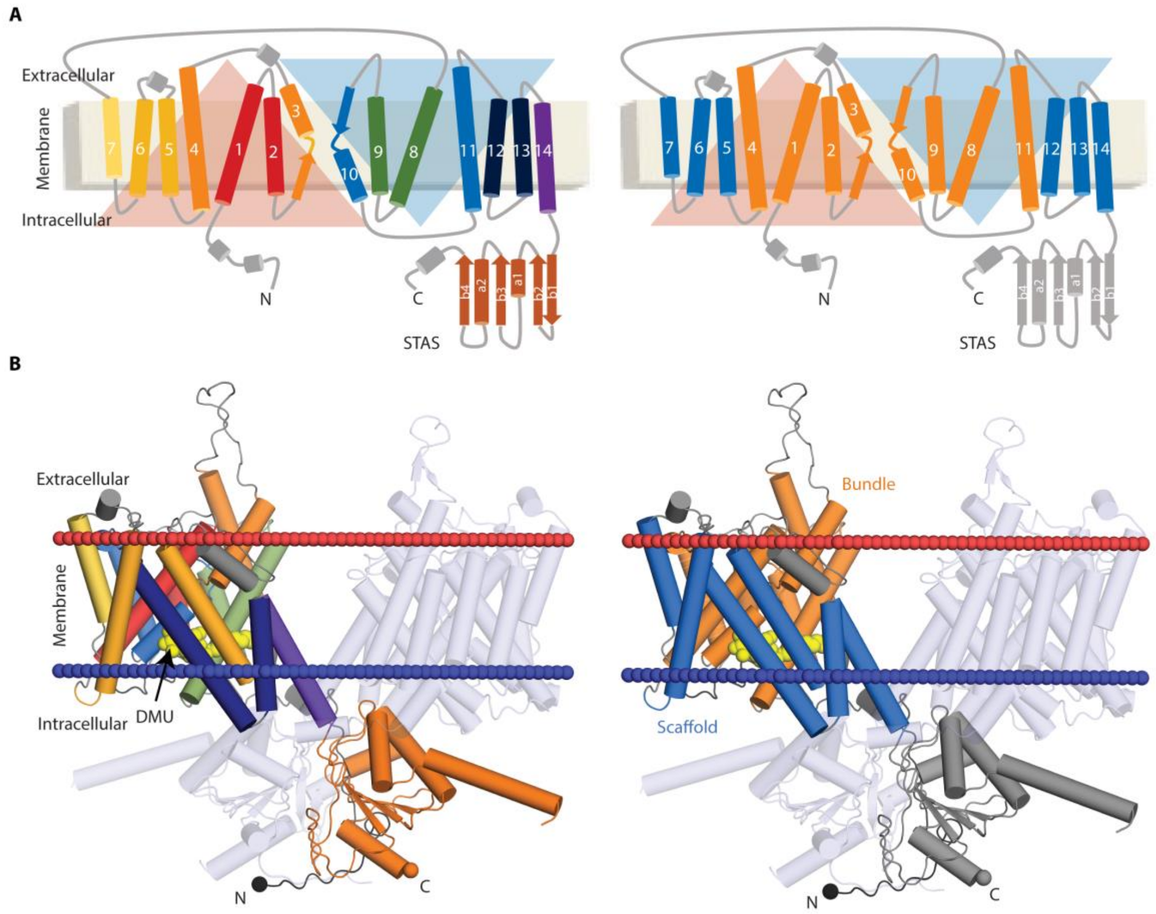

Fig. S2. Topology of SLC26A4 and structural model of mouse SLC26A4.

(**A**) SLC26A4 membrane topology derived from the cryo-EM structure of mouse SLC26A4 (PDBid: 7wk1) (*7*). Each monomer of SLC26A4 contains an intracytoplasmic amino-terminal domain, a transmembrane domain with 14 transmembrane helices (TMs) and an intracellular STAS domain at the carboxy-terminus. The two inverted repeat units present in SLC26A4 are indicated as two triangles (red for repeat unit 1 (RU1) and blue for repeat unit 2 (RU2)). The membrane is shown as a horizontal cream bar and each TM helix is indicated with a bar. On the left panel, TMs from the first repeat unit (TM1-7) are colored red, orange and yellow while those from the second repeat unit (TM 8-14) are colored in green, light and dark blue and purple. On the right panel, the helices of the scaffold are colored in orange, whereas the helices of the bundle are blue. (**B**) Structural cartoon model of mSLC26A4 dimer after the remodeling of the loop containing the 536-YKNL tyrosine-based motif. Only one monomer is color-coded according to the corresponding membrane topology in (**A**). The membrane is represented by two sphere planes indicating extracellular (red) and intracellular (blue) lipid leaflets. The central binding site for each monomer is occupied by a molecule of DMU (Decyl-Beta-D-Maltopyranoside) represented as yellow spheres. The coordinates of DMU were obtained after structurally superimposing mSLC26A4 model to the structure of SLC26A4 prokaryotic homolog *Deinococcus geothermalis* SLC26Dg (PDB id: 5da0) (*75*).

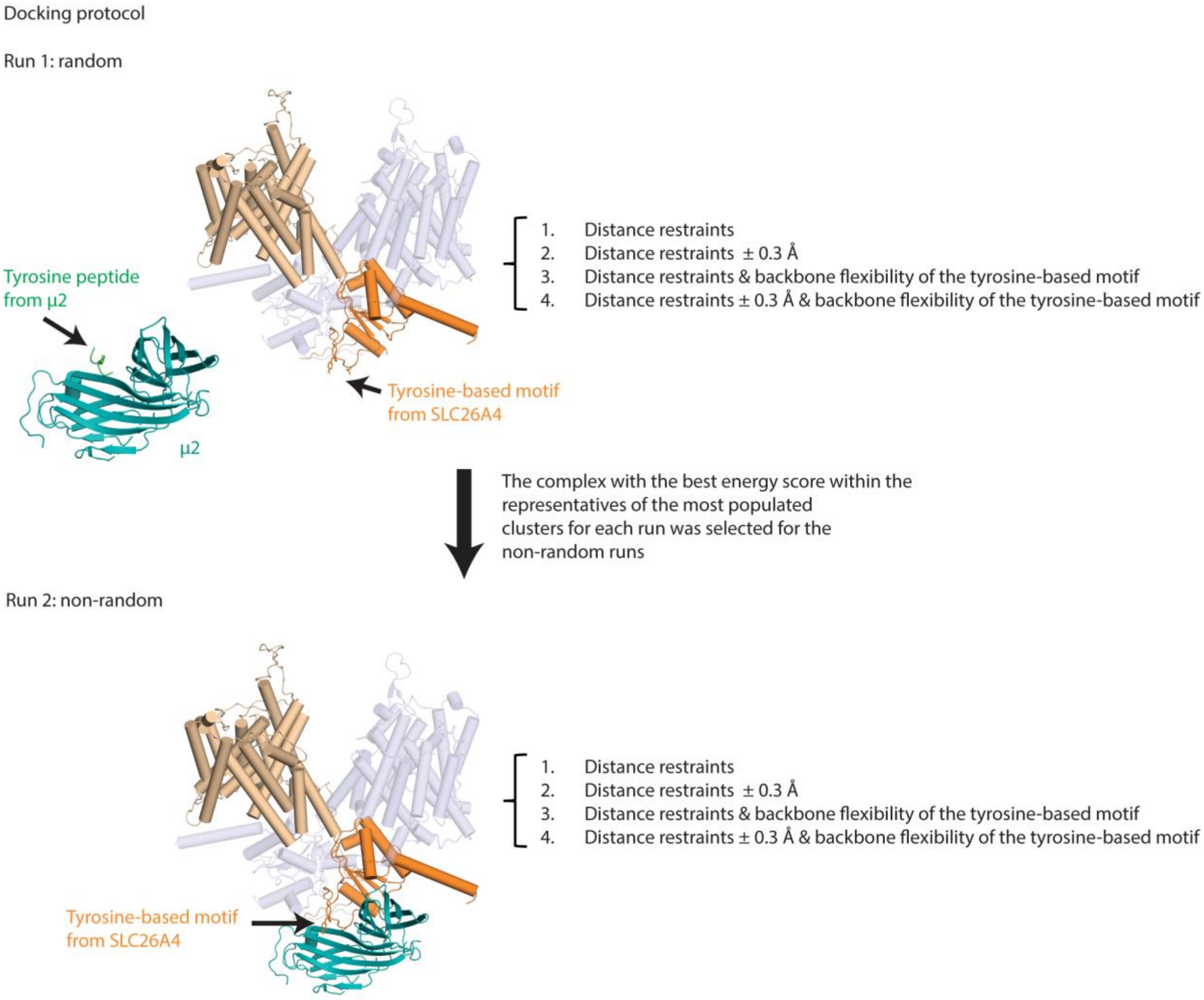

Fig. S3. Flow chart of the protein-protein docking protocol.
Eight docking runs were used to obtain the mouse SLC26A4-μ2 complex. These can be divided into two groups. In the first one, the relative orientation between SLC26A4 and μ2 was randomized as per standard procedure of HADDOCK, while in the second one the initial relative position between the two proteins was that derived from the best model from run1. In both groups, four docking runs using different conditions were performed. In all of them distance restraints derived from the X-ray of μ2 bound to a tyrosine peptide (PDBid: 6bnt (*29*)) (table S2) were applied and only in two of them a deviation of ± 0.3 Å in those distances was allowed. Backbone flexibility for the residues of the tyrosine-based motif of SLC26A4 was implemented in two of the runs.

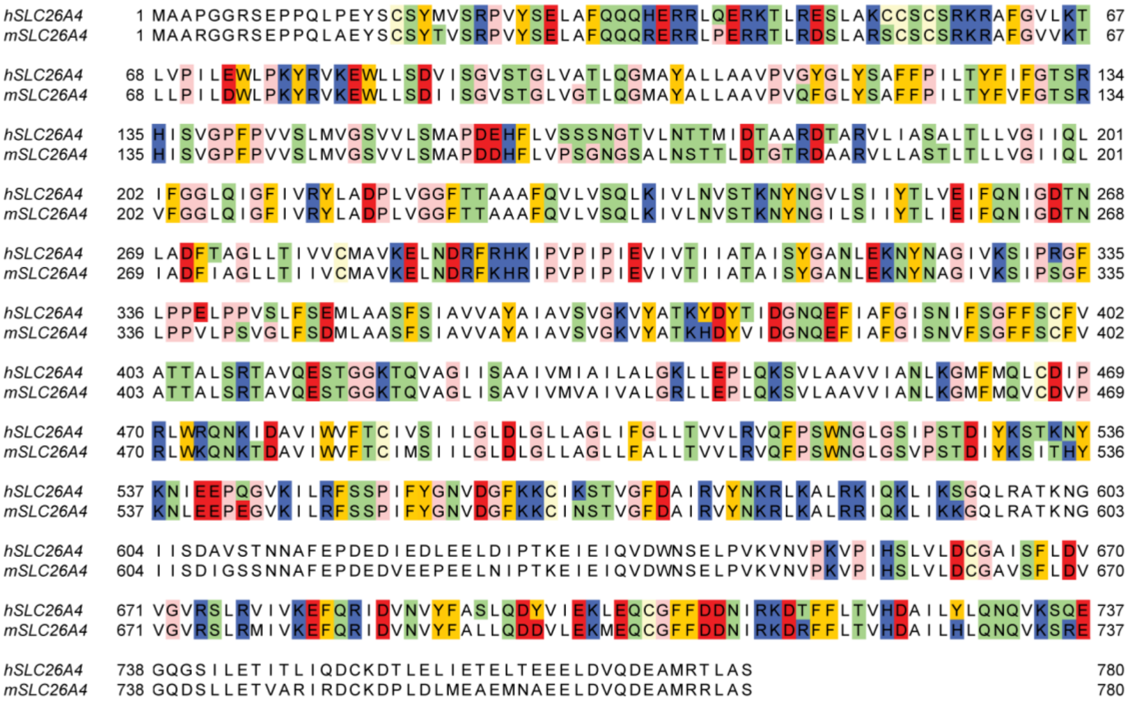

Fig. S4. Amino-acid sequence alignment used to generate the structural model of human SLC26A4 using mouse SLC26A4 as template.

Amino-acid residues of human (h) and mouse (m) SLC26A4 are colored as follows: white: hydrophobic, green: polar, blue: positively charged, red: negatively charged, orange: aromatic, pink: proline and glycine.

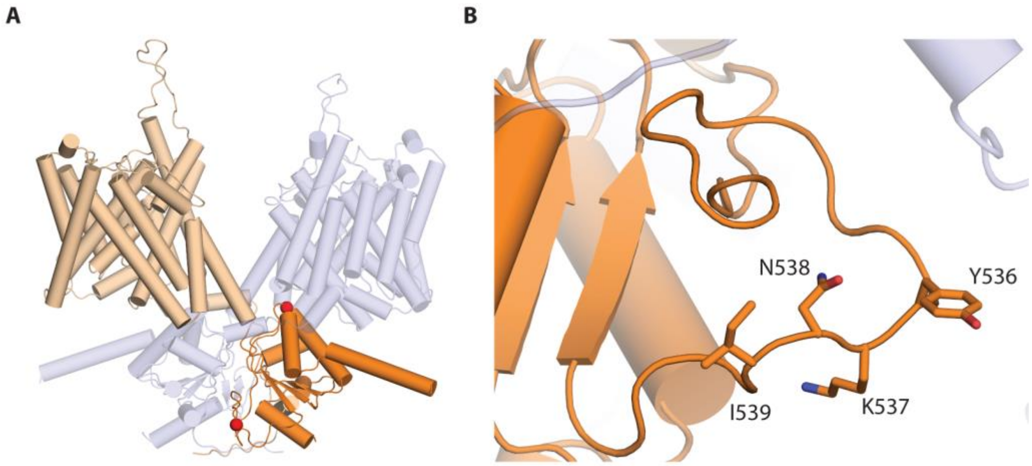

Fig. S5. Identification of the tyrosine binding motifs of μ2 in human SLC26A4 model. Structural model of human SLC26A4 shown in cartoon where one monomer is highlighted in orange. The transmembrane and STAS domains are represented in light and dark orange, respectively. (**A**) Two tyrosine-based motifs are present in the STAS containing carboxy-terminal domain. The C-α atom of the tyrosine residue in each motif is indicated with a red sphere. (**B**) Close-up view of the tyrosine-based motif 536-YKNI present in a flexible loop exposed to the solvent and accessible to potential interactions. The four residues of the tyrosine-based motif are shown as orange sticks where the oxygen and nitrogen atoms are colored in red and blue respectively.

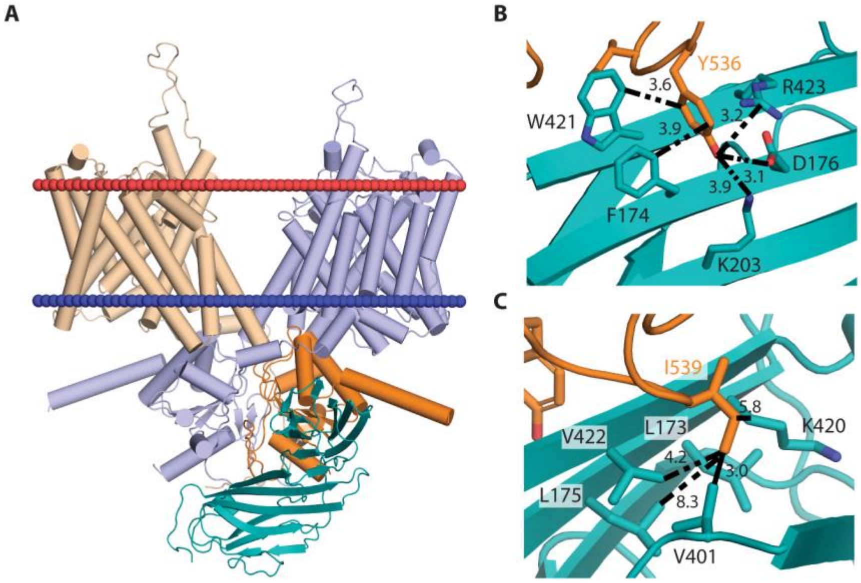

Fig. S6. Structural model of human SLC26A4 complexed with μ2 subunit of AP-2.
(**A**) The structural model of human SLC26A4 complexed with the μ2 subunit of AP-2 is shown with the membrane represented as in fig. S2. The monomers of SLC26A4 are colored in orange and blue, while the μ subunit of AP-2 is colored in emerald-green. (**B**, **C**) Close-up views of the binding sites in μ2 for the tyrosine (Y536) and hydrophobic residue (I539) within the tyrosine-based motif of human SLC26A4. Residues taking part in hydrogen bond or hydrophobic interactions are shown as sticks and the interactions are indicated as dashed lines. Heteroatoms nitrogen and oxygen are colored in blue and red, respectively.

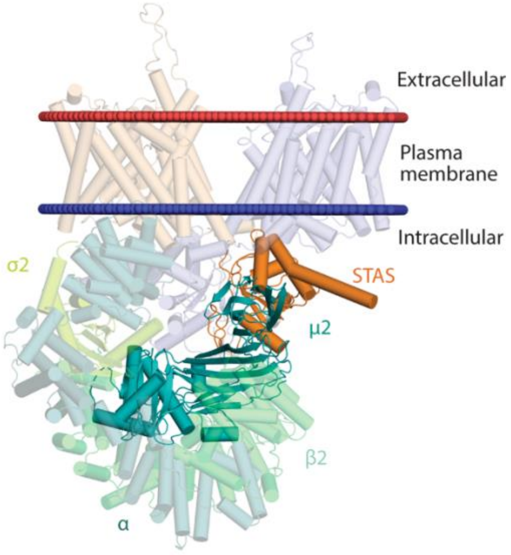

Fig. S7. Structural model of human SLC26A4 complexed with AP-2.
The structural model of human SLC26A4 in complex with AP-2 was obtained after structural superimposition of μ2 subunit in the SLC26A4-μ2 complex and that of the X-ray structure of *Rattus norvegicus* AP-2 in open form (PDBid: 2xa7 (*35*)). The monomers of SLC26A4 are colored in orange and blue while AP-2 subunits are colored in different shades of green.

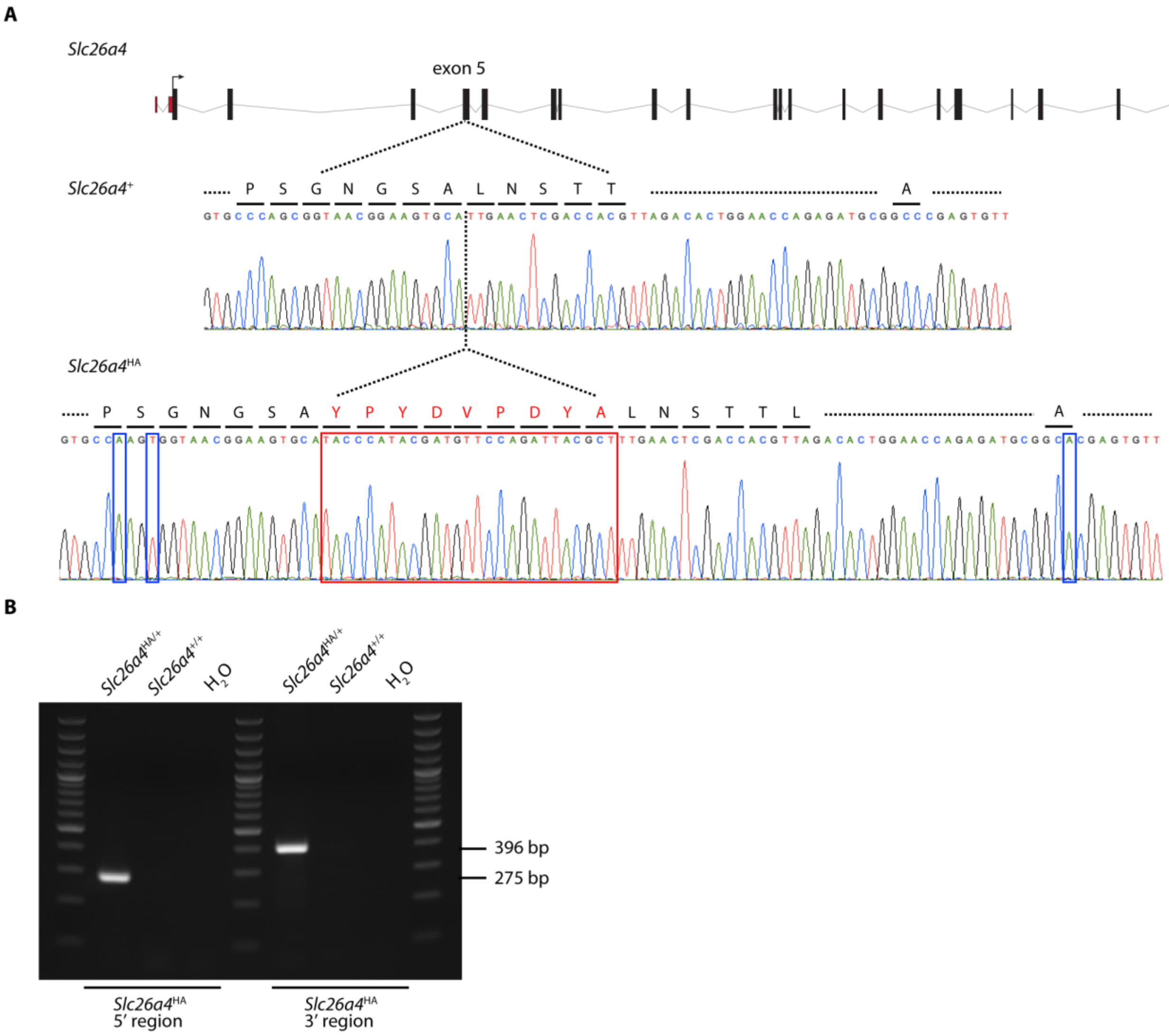

Fig. S8. Generation of *Slc26a4*$^{HA/+}$ knock in mice.

(**A**) Nucleotide sequence of *Slc26a4*^{HA} allele in chromosome 12 showing the insertion site of the coding sequence for HA tag (red rectangle) within exon 5 of *Slc26a4* gene. Three additional variants introduced during the process of CRISPR-Cas9 editing and expected to lead to synonymous changes are shown in blue rectangles. (**B**) Genotyping of *Slc26a4*$^{HA/+}$ mice by PCR. Amplicons of 275 and 396 bp indicating the presence of the coding sequence for HA tag at the expected locus, are only detected in the genomic DNA of *Slc26a4*$^{HA/+}$ mice but not in the genomic DNA of their wild-type littermates. The primers used are reported in table S4. H_2O: negative control without DNA template. DNA size marker (100 bp ladder, SM0323, Thermo Fisher Scientific).

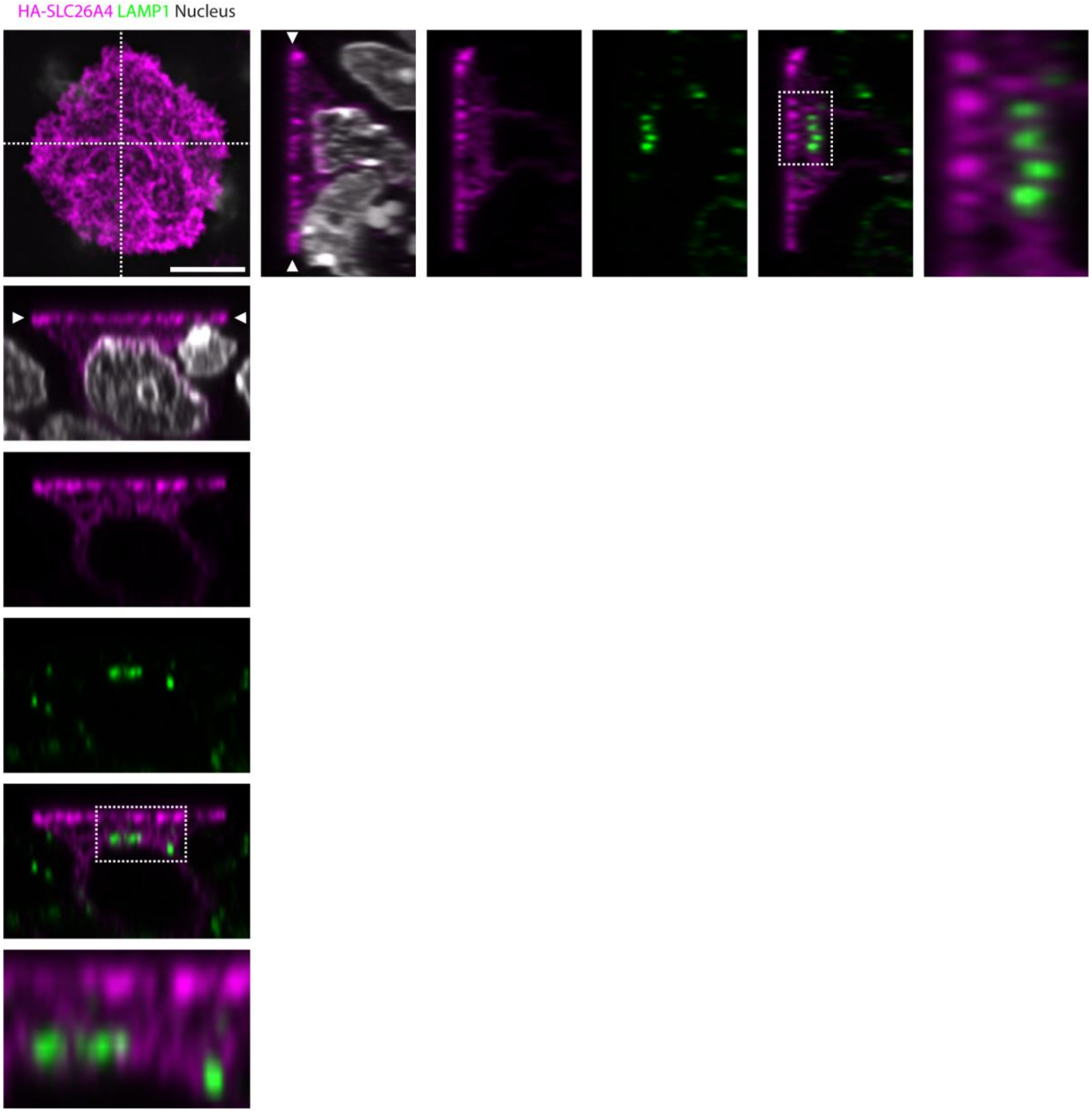

Fig. S9. High magnification images of a mitochondria rich cell from E16.5 *Slc26a4*$^{HA/+}$ mouse labeled with anti-HA antibodies in permeabilizing conditions.

Confocal images obtained from whole-mount opened endolymphatic sac labeled with antibodies recognizing HA tag to detect HA-SLC26A4 (magenta), Hoechst 33342 to visualize the cell nuclei (white), and with antibodies recognizing the Lysosomal Associated Membrane Protein 1 (LAMP1), a lysosomal marker (green, rat anti-LAMP1, dilution 1:500, #1D4B, Developmental Studies Hybridoma Bank). Tissue cross-sections in the three perpendicular planes at the levels of the white dotted lines and arrow heads showing HA-SLC26A4 labeling in the cytoplasm of this cell. HA-SLC26A4 labeling is strongly enriched in the apical region of the cell (at the level of the arrow heads), which may correspond to HA-SLC26A4 present at the plasma membrane or

associated with some of the vesicles localized just below it. HA-SLC26A4 labeling overlaps with LAMP1 labeling in the region just above the nucleus of this cell, as also shown as higher magnification in the lowest, and right panels. Scale bar: 5 µm.

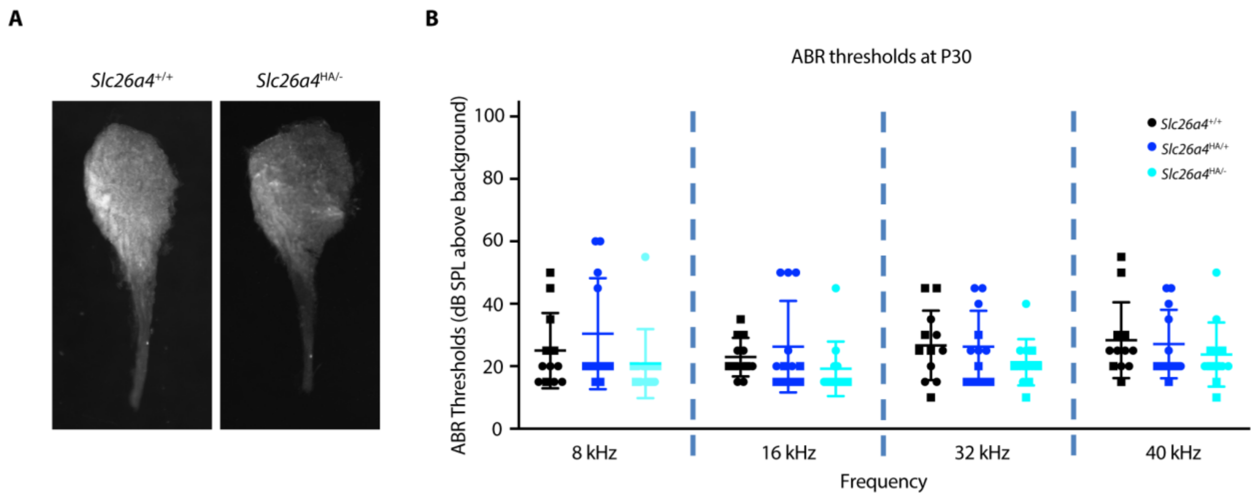

Fig. S10. Mice carrying *Slc26a4*^{HA} allele do not show enlarged endolymphatic sac and have similar hearing thresholds as their wild-type littermates.

(**A**) Representative images of endolymphatic sacs from *Slc26a4*$^{\mathrm{HA/-}}$ and *Slc26a4*$^{+/+}$ littermate mice at P0. An enlargement of the endolymphatic sac or duct was not detected. A total of 6 endolymphatic sacs from 3 mice of each genotype, obtained from 3 litters, were microdissected and compared. (**B**) Auditory brainstem responses (ABRs) of *Slc26a4*$^{+/+}$, *Slc26a4*$^{\mathrm{HA/+}}$ and *Slc26a4*$^{\mathrm{HA/-}}$ littermates were measured at P30 to assess auditory function in mice of these different genotypes. For each genotype 3 mice of each sex were studied, ABR thresholds for each of their ears were included and considered as different measures rather than technical replicates from the same sample as hearing loss associated with *SLC26A4* pathogenic variants can be asymmetric in both human and mouse (*10*). Results are presented as mean ± SD. No significant differences were identified between values obtained for *Slc26a4*$^{\mathrm{HA/+}}$ and *Slc26a4*$^{\mathrm{HA/-}}$ mice as compared to the control group of *Slc26a4*$^{+/+}$ mice, using two-way ANOVA followed by Tukey's multiple comparisons tests. Squares and dots correspond to measurements obtained in females and males, respectively. For each genotype, a few ears had elevated thresholds in the 40 to 60 dB range. We did not detect middle ear infection in these ears with otoscopy, but it could have been missed.

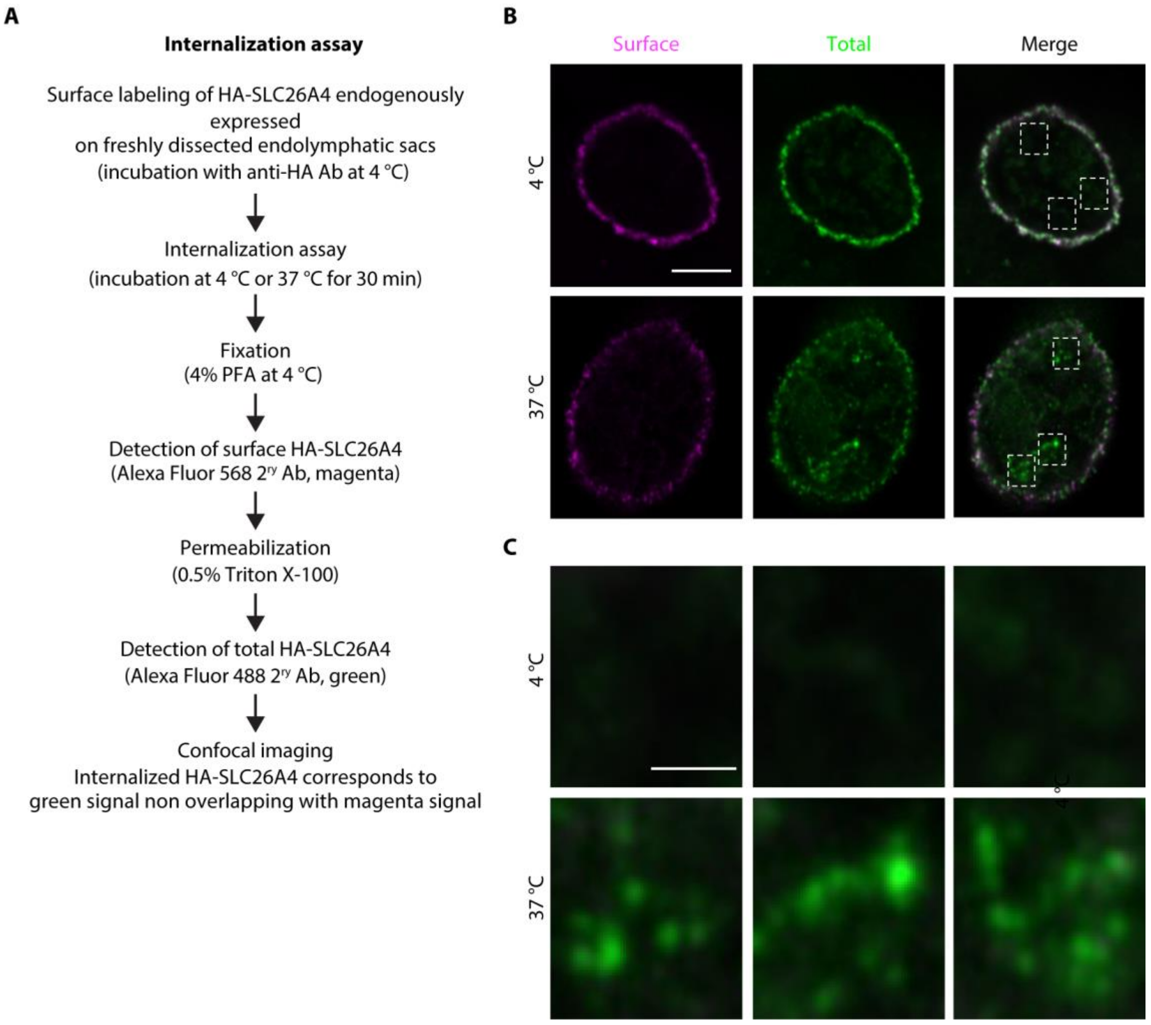

Fig. S11. Internalization assay of endogenously expressed HA-SLC26A4 in MRCs of freshly dissected endolymphatic sacs.

(**A**) Experimental approach of this internalization assay. Ab, antibodies. (**B**) Single-plane confocal images of MRCs from E16.5 *Slc26a4*$^{HA/+}$ mouse endolymphatic sacs incubated at 4°C or 37°C following preincubation with anti-HA antibodies. Surface HA-SLC26A4 was labeled with Alexa Fluor 568-conjugated secondary antibodies (magenta) in non-permeabilizing conditions. Total HA-SLC26A4 signal was detected with Alexa Fluor 488-conjugated secondary antibodies after permeabilization (green). HA-SLC26A4 was detected in the cytoplasm of the cells incubated at 37°C but not in the cells incubated at 4°C. (**C**) Higher magnification of the cytoplasmic regions indicated in the merge panel in B. Scale bars: 5 µm (B), 1 µm (C).

S26A1	528	F	E	G	L	531
S26A2	569	Y	K	N	L	572
S26A3	526	Y	Y	D	M	529
S26A4	536	Y	K	N	I	539
S26A5	526	Y	E	E	V	529
S26A6	531	Y	S	E	A	534
S26A7	494	-	M	D	S	496
S26A8	544	Y	R	E	I	547
S26A9	520	Y	N	R	A	523
S2610		-	-	-	-	
S2611		-	-	-	-	

Fig. S12. Conservation of the tyrosine-based motif in the cytosolic carboxy-terminal domain of human SLC26A4 necessary for its interaction with μ2 within SLC26 family members. Conservation across the SLC26 family members of the residues forming the tyrosine-based motif identified as necessary for SLC26A4 to interact with μ2. The amino-acid sequences obtained through Uniprot server (http://www.uniprot.org) were analyzed in Jalview. Alignment was performed using ClustalW algorithm (*56*). Protein sequences identifiers for human SLC26 family members are *Homo sapiens*: Q9H2B4 for SLC26A1, P50443 for SLC26A2, P40879 for SLC26A3, O43511 for SLC26A4, P58743 for SLC26A5, Q9BXS9 for SLC26A6, Q8TE54 for SLC26A7, Q96RN1 for SLC26A8, Q7LBE3 for SLC26A9, Q8NG04 for SLC26A10, Q86WA9 for SLC26A11. Physico-chemical properties of the residues are color coded as follows: hydrophobic (white), aromatic (orange), positively charged (blue), negatively charged (red), polar (green), glycine (pink).

**Expression construct**	**Vector (backbone)**	**Insert (amino acids or reference)**
pEGFP-MYO10	pEGFP-C3	MYO10$^{\mathrm{NO\ BAIT}}$ (*26*)
pmCherry-MYO10	pmCherry-C1	MYO10$^{\mathrm{NO\ BAIT}}$ (*26*)
pmCherry-MYO10-SLC26A4-Ct^{BAIT}	pmCherry-MYO10	SLC26A4-Ct (512-780)
pμ2 MID-EGFP^{PREY}	pEGFP-N1	μ2-MID (124-435)
pμ2 FL-EGFP^{PREY}	pEGFP-N1	μ2-FL (1-435)
pμ2 Ct-EGFP^{PREY}	pEGFP-N1	μ2-Ct (170-435)
pEGFP-MYO10-μ2-MID^{BAIT}	pEGFP-MYO10	μ2-MID (124-435)
pEGFP-MYO10-μ2-FL^{BAIT}	pEGFP-MYO10	μ2-FL (1-435)
pmCherry-SLC26A4-Ct^{PREY}	pmCherry-C1	SLC26A4-Ct (512-780)
pmCherry-MYO10-SLC26A4-Ct (Y530A, Y536A, Y556A, Y691A)^{BAIT}	pmCherry-MYO10	SLC26A4-Ct (512-780)
pmCherry-MYO10-SLC26A4-Ct (Y530A, Y556A, Y691A)^{BAIT}	pmCherry-MYO10	SLC26A4-Ct (512-780)
pmCherry-MYO10-SLC26A4-Ct (Y536A)^{BAIT}	pmCherry-MYO10	SLC26A4-Ct (512-780)
pmCherry-MYO10-SLC26A4-Ct (L539A)^{BAIT}	pmCherry-MYO10	SLC26A4-Ct (512-780)
pμ2 MID (D176A)-EGFP^{PREY}	pEGFP-N1	μ2-MID (124-435)
pCMV-HA-SLC26A4	pCMV	SLC26A4 (1-780)

Table S1. List of plasmids and constructs used in NanoSPD assays
The amino-acids sequences of the inserts are indicated using reference sequences NP_035997.1 for mouse SLC26A4 and NP_033809.1 for mouse μ2. Myosin-10 heavy meromyosin-like domain (MYO10^{HMM}) is designated here as MYO10.

Cα atom	Cα atom	Distance (Å)
Y536	F174	8
	D176	9
	K203	12
	R423	5
L539	L175	9
	V401	8
	K420	5
	V422	8

Table S2. List of distance restraints used during the docking runs for mouse SLC26A4 and μ2.

	Amplicon size (bp)	
Sequences of the primers	*Slc26a4*$^{+}$	*Slc26a4*^{HA}
5'- GCCTTGAGACATCTGCTTCTCAGCCTC-3'<br>5'- ATTCATGTCTCCTGCACCCGTTCCATGC-3'	606	633

Table S3. Sequences of the primers designed to amplify and Sanger sequence the region targeted by CRISPR/Cas9 editing and identify the desired allele

Amplicons were generated by PCR using the GoTaq Flexi DNA Polymerase (Promega Corp). PCR was carried out using one cycle of denaturation at 94°C for 3 min, 35 cycles of denaturation at 94°C for 30 s, annealing at 57°C for 30 s, and extension at 72°C for 40 s, followed by a final extension at 72°C for 10 min.

Allele	Sequences of the genotyping primers	Amplicon size (bp)
Slc26a4^{HA}<br>*5' region*	5'-GCCTTGAGACATCTGCTTCTCAGCCTC-3'<br>5'-gttcaAAGCGTAATCTGGAACATCGTA-3'	275
Slc26a4^{HA}<br>*3' region*	5'- gtgcaTACCCATACGATGTTCCAG-3'<br>5'- ATTCATGTCTCCTGCACCCGTTCCATGC-3'	396

Table S4. Sequences of the primers designed to detect *Slc26a4*^{HA} and PCR conditions used for the genotyping of this mouse line

After initial identification of the allele obtained by CRISPR/Cas9 using PCR and sequencing according to table S3, allele specific primers were designed to identify the *Slc26a4*^{HA} allele and used for subsequent genotyping. PCRs were carried out using Go Taq Flexi Polymerase (Promega), using one cycle of denaturation at 94°C for three min, 35 cycles of denaturation at 94°C for 30 s, annealing at 57°C for 30 s, and extension at 72°C for 30 s, followed by a final extension at 72°C for 10 min.

Other Supplementary Materials for this manuscript include the following:

Data file S1.
Data organized by figure and panel.

Structural models of mouse and human SLC26A4-μ2 obtained using *in silico* molecular modelling and protein-protein docking techniques.
These models are available in the following files: mSLC26A4_mMu2_complex.pdb (mouse) and hSLC26A4_hMu2_complex.pdb (human).

File used to obtain the structural models of mouse and human SLC26A4 complexed with AP-2.
AP-2 complex with the atomic coordinates after the μ2 subunit of the complex was structurally superimposed to that in the SLC26A4 complex. For visualization as in Fig. 7 and fig. S7 open this file with the file corresponding to human or mouse SLC26A4-μ2 structural model: 2xa7_supperimposed_to_SLC26A4_Mu2_complex.pdb.

REFERENCES AND NOTES

1. A. J. Griffith, P. Wangemann, Hearing loss associated with enlargement of the vestibular aqueduct: Mechanistic insights from clinical phenotypes, genotypes, and mouse models. *Hear. Res.* **281**, 11–17 (2011).

2. A. Dahlmann, M. von During, The endolymphatic duct and sac of the rat: A histological, ultrastructural, and immunocytochemical investigation. *Cell Tissue Res.* **282**, 277–289 (1995).

3. K. Honda, S. H. Kim, M. C. Kelly, J. C. Burns, L. Constance, X. Li, F. Zhou, M. Hoa, M. W. Kelley, P. Wangemann, R. J. Morell, A. J. Griffith, Molecular architecture underlying fluid absorption by the developing inner ear. *eLife* **6**, e26851 (2017).

4. L. A. Everett, B. Glaser, J. C. Beck, J. R. Idol, A. Buchs, M. Heyman, F. Adawi, E. Hazani, E. Nassir, A. D. Baxevanis, V. C. Sheffield, E. D. Green, Pendred syndrome is caused by mutations in a putative sulphate transporter gene (PDS). *Nat. Genet.* **17**, 411–422 (1997).

5. L. M. Luxon, M. Cohen, R. A. Coffey, P. D. Phelps, K. E. Britton, H. Jan, R. C. Trembath, W. Reardon, Neuro-otological findings in Pendred syndrome. *Int. J. Audiol.* **42**, 82–88 (2003).

6. W. Reardon, R. C. Trembath, Pendred syndrome. *J. Med. Genet.* **33**, 1037–1040 (1996).

7. Q. Liu, X. Zhang, H. Huang, Y. Chen, F. Wang, A. Hao, W. Zhan, Q. Mao, Y. Hu, L. Han, Y. Sun, M. Zhang, Z. Liu, G. L. Li, W. Zhang, Y. Shu, L. Sun, Z. Chen, Asymmetric pendrin homodimer reveals its molecular mechanism as anion exchanger. *Nat. Commun.* **14**, 3012 (2023).

8. P. Wangemann, K. Nakaya, T. Wu, R. J. Maganti, E. M. Itza, J. D. Sanneman, D. G. Harbidge, S. Billings, D. C. Marcus, Loss of cochlear HCO_3- secretion causes deafness via endolymphatic acidification and inhibition of Ca^{2+} reabsorption in a Pendred syndrome mouse model. *Am. J. Physiol. Renal Physiol.* **292**, F1345–F1353 (2007).

9. H. M. Kim, P. Wangemann, Epithelial cell stretching and luminal acidification lead to a retarded development of stria vascularis and deafness in mice lacking pendrin. *PLOS ONE* **6**, e17949 (2011).

10. B. Y. Choi, H. M. Kim, T. Ito, K. Y. Lee, X. Li, K. Monahan, Y. Wen, E. Wilson, K. Kurima, T. L. Saunders, R. S. Petralia, P. Wangemann, T. B. Friedman, A. J. Griffith, Mouse model of enlarged vestibular aqueducts defines temporal requirement of *Slc26a4* expression for hearing acquisition. *J. Clin. Invest.* **121**, 4516–4525 (2011).

11. H. M. Kim, P. Wangemann, Failure of fluid absorption in the endolymphatic sac initiates cochlear enlargement that leads to deafness in mice lacking pendrin expression. *PLOS ONE* **5**, e14041 (2010).

12. X. Li, J. D. Sanneman, D. G. Harbidge, F. Zhou, T. Ito, R. Nelson, N. Picard, R. Chambrey, D. Eladari, T. Miesner, A. J. Griffith, D. C. Marcus, P. Wangemann, SLC26A4 targeted to the endolymphatic sac rescues hearing and balance in *Slc26a4* mutant mice. *PLOS Genet.* **9**, e1003641 (2013).

13. K. Ishii, I. Norota, Y. Obara, Endocytic regulation of voltage-dependent potassium channels in the heart. *J. Pharmacol. Sci.* **120**, 264–269 (2012).

14. M. Kaksonen, A. Roux, Mechanisms of clathrin-mediated endocytosis. *Nat. Rev. Mol. Cell Biol.* **19**, 313–326 (2018).

15. K. Kurakami, I. Norota, F. Nasu, S. Ohshima, Y. Nagasawa, Y. Konno, Y. Obara, K. Ishii, KCNQ1 is internalized by activation of α_1 adrenergic receptors. *Biochem. Pharmacol.* **169**, 113628 (2019).

16. R. A. Shimkets, R. P. Lifton, C. M. Canessa, The activity of the epithelial sodium channel is regulated by clathrin-mediated endocytosis. *J. Biol. Chem.* **272**, 25537–25541 (1997).

17. N. A. Bradbury, J. A. Cohn, C. J. Venglarik, R. J. Bridges, Biochemical and biophysical identification of cystic fibrosis transmembrane conductance regulator chloride channels as components of endocytic clathrin-coated vesicles. *J. Biol. Chem.* **269**, 8296–8302 (1994).

18. B. M. Collins, A. J. McCoy, H. M. Kent, P. R. Evans, D. J. Owen, Molecular architecture and functional model of the endocytic AP2 complex. *Cell* **109**, 523–535 (2002).

19. D. J. Owen, P. R. Evans, A structural explanation for the recognition of tyrosine-based endocytotic signals. *Science* **282**, 1327–1332 (1998).

20. H. Ohno, M. C. Fournier, G. Poy, J. S. Bonifacino, Structural determinants of interaction of tyrosine-based sorting signals with the adaptor medium chains. *J. Biol. Chem.* **271**, 29009–29015 (1996).

21. W. Boll, H. Ohno, Z. Songyang, I. Rapoport, L. C. Cantley, J. S. Bonifacino, T. Kirchhausen, Sequence requirements for the recognition of tyrosine-based endocytic signals by clathrin AP-2 complexes. *EMBO J.* **15**, 5789–5795 (1996).

22. H. Ohno, J. Stewart, M. C. Fournier, H. Bosshart, I. Rhee, S. Miyatake, T. Saito, A. Gallusser, T. Kirchhausen, J. S. Bonifacino, Interaction of tyrosine-based sorting signals with clathrin-associated proteins. *Science* **269**, 1872–1875 (1995).

23. Y. H. Kim, T. H. Kwon, S. Frische, J. Kim, C. C. Tisher, K. M. Madsen, S. Nielsen, Immunocytochemical localization of pendrin in intercalated cell subtypes in rat and mouse kidney. *Am. J. Physiol. Renal Physiol.* **283**, F744–F754 (2002).

24. I. E. Royaux, S. M. Wall, L. P. Karniski, L. A. Everett, K. Suzuki, M. A. Knepper, E. D. Green, Pendrin, encoded by the Pendred syndrome gene, resides in the apical region of renal intercalated cells and mediates bicarbonate secretion. *Proc. Natl. Acad. Sci. U.S.A.* **98**, 4221–4226 (2001).

25. S. M. Wall, Y. H. Kim, L. Stanley, D. M. Glapion, L. A. Everett, E. D. Green, J. W. Verlander, NaCl restriction upregulates renal *Slc26a4* through subcellular redistribution: Role in Cl- conservation. *Hypertension* **44**, 982–987 (2004).

26. J. E. Bird, M. Barzik, M. C. Drummond, D. C. Sutton, S. M. Goodman, E. L. Morozko, S. M. Cole, A. K. Boukhvalova, J. Skidmore, D. Syam, E. A. Wilson, T. Fitzgerald, A. U. Rehman, D. M. Martin, E. T. Boger, I. A. Belyantseva, T. B. Friedman, Harnessing molecular motors for nanoscale pulldown in live cells. *Mol. Biol. Cell* **28**, 463–475 (2017).

27. T. A. Peters, E. L. Tonnaer, W. Kuijpers, C. W. Cremers, J. H. Curfs, Differences in endolymphatic sac mitochondria-rich cells indicate specific functions. *Laryngoscope* **112**, 534–541 (2002).

28. D. Runggaldier, L. G. Pradas, P. H. Neckel, A. F. Mack, B. Hirt, C. Gleiser, Claudin expression in the rat endolymphatic duct and sac—First insights into regulation of the paracellular barrier by vasopressin. *Sci. Rep.* **7**, 45482 (2017).

29. E. Choi, S. Kikuchi, H. Gao, K. Brodzik, I. Nassour, A. Yopp, A. G. Singal, H. Zhu, H. Yu, Mitotic regulators and the SHP2-MAPK pathway promote IR endocytosis and feedback regulation of insulin signaling. *Nat. Commun.* **10**, 1473 (2019).

30. J. S. Bonifacino, L. M. Traub, Signals for sorting of transmembrane proteins to endosomes and lysosomes. *Annu. Rev. Biochem.* **72**, 395–447 (2003).

31. K. F. Johnson, W. Chan, S. Kornfeld, Cation-dependent mannose 6-phosphate receptor contains two internalization signals in its cytoplasmic domain. *Proc. Natl. Acad. Sci. U.S.A.* **87**, 10010–10014 (1990).

32. B. Webb, A. Sali, Comparative protein structure modeling using MODELLER. *Curr. Protoc. Bioinformatics* **54**, 5.6.1–5.6.37 (2016).

33. R. V. Honorato, P. I. Koukos, B. Jimenez-Garcia, A. Tsaregorodtsev, M. Verlato, A. Giachetti, A. Rosato, A. Bonvin, Structural biology in the clouds: The WeNMR-EOSC ecosystem. *Front. Mol. Biosci.* **8**, 729513 (2021).

34. G. C. P. van Zundert, J. Rodrigues, M. Trellet, C. Schmitz, P. L. Kastritis, E. Karaca, A. S. J. Melquiond, M. van Dijk, S. J. de Vries, A. Bonvin, The HADDOCK2.2 web server: User-friendly integrative modeling of biomolecular complexes. *J. Mol. Biol.* **428**, 720–725 (2016).

35. L. P. Jackson, B. T. Kelly, A. J. McCoy, T. Gaffry, L. C. James, B. M. Collins, S. Honing, P. R. Evans, D. J. Owen, A large-scale conformational change couples membrane recruitment to cargo binding in the AP2 clathrin adaptor complex. *Cell* **141**, 1220–1229 (2010).

36. K. Isgrig, Y. Ishibashi, H. J. Lee, J. Zhu, M. Grati, J. Bennett, A. J. Griffith, I. Roux, W. W. Chien, AAV8BP2 and AAV8 transduce the mammalian cochlear lateral wall and endolymphatic sac with high efficiency. *Mol. Ther. Methods Clin. Dev.* **26**, 371–383 (2022).

37. E. Macia, M. Ehrlich, R. Massol, E. Boucrot, C. Brunner, T. Kirchhausen, Dynasore, a cell-permeable inhibitor of dynamin. *Dev. Cell* **10**, 839–850 (2006).

38. A. Young, M. Gentzsch, C. Y. Abban, Y. Jia, P. I. Meneses, R. J. Bridges, N. A. Bradbury, Dynasore inhibits removal of wild-type and ΔF508 cystic fibrosis transmembrane conductance regulator (CFTR) from the plasma membrane. *Biochem. J.* **421**, 377–385 (2009).

39. T. Cronqvist, L. Erlandsson, D. Tannetta, S. R. Hansson, Placental syncytiotrophoblast extracellular vesicles enter primary endothelial cells through clathrin-mediated endocytosis. *Placenta* **100**, 133–141 (2020).

40. T. Kirchhausen, E. Macia, H. E. Pelish, Use of dynasore, the small molecule inhibitor of dynamin, in the regulation of endocytosis. *Methods Enzymol.* **438**, 77–93 (2008).

41. G. Auciello, D. L. Cunningham, T. Tatar, J. K. Heath, J. Z. Rappoport, Regulation of fibroblast growth factor receptor signalling and trafficking by Src and Eps8. *J. Cell Sci.* **126**, 613–624 (2013).

42. S. Rizzolio, L. Tamagnone, Antibody-feeding assay: A method to track the internalization of neuropilin-1 and other cell surface receptors. *Methods Mol. Biol.* **1493**, 311–319 (2017).

43. K. Wasano, S. Takahashi, S. K. Rosenberg, T. Kojima, H. Mutai, T. Matsunaga, K. Ogawa, K. Homma, Systematic quantification of the anion transport function of pendrin (*SLC26A4*) and its disease-associated variants. *Hum. Mutat.* **41**, 316–331 (2020).

44. V. C. S. de Moraes, E. Bernardinelli, N. Zocal, J. A. Fernandez, C. Nofziger, A. M. Castilho, E. L. Sartorato, M. Paulmichl, S. Dossena, Reduction of cellular expression levels is a common feature of functionally affected pendrin (*SLC26A4*) protein variants. *Mol. Med.* **22**, 41–53 (2016).

45. B. Y. Choi, A. K. Stewart, A. C. Madeo, S. P. Pryor, S. Lenhard, R. Kittles, D. Eisenman, H. J. Kim, J. Niparko, J. Thomsen, K. S. Arnos, W. E. Nance, K. A. King, C. K. Zalewski, C. C. Brewer, T. Shawker, J. C. Reynolds, J. A. Butman, L. P. Karniski, S. L. Alper, A. J. Griffith, Hypo-functional *SLC26A4* variants associated with nonsyndromic hearing loss and enlargement of the vestibular aqueduct: Genotype-phenotype correlation or coincidental polymorphisms? *Hum. Mutat.* **30**, 599–608 (2009).

46. P. Chattaraj, T. Munjal, K. Honda, N. D. Rendtorff, J. S. Ratay, J. A. Muskett, D. S. Risso, I. Roux, E. M. Gertz, A. A. Schaffer, T. B. Friedman, R. J. Morell, L. Tranebjaerg, A. J. Griffith, A common *SLC26A4*-linked haplotype underlying non-syndromic hearing loss with enlargement of the vestibular aqueduct. *J. Med. Genet.* **54**, 665–673 (2017).

47. J. Xu, S. Barone, M. Varasteh Kia, L. S. Holliday, K. Zahedi, M. Soleimani, Identification of IQGAP1 as a SLC26A4 (Pendrin)-binding protein in the kidney. *Front. Mol. Biosci.* **9**, 874186 (2022).

48. J. S. Yoon, H. J. Park, S. Y. Yoo, W. Namkung, M. J. Jo, S. K. Koo, H. Y. Park, W. S. Lee, K. H. Kim, M. G. Lee, Heterogeneity in the processing defect of *SLC26A4* mutants. *J. Med. Genet.* **45**, 411–419 (2008).

49. G. Tamma, S. Dossena, Functional interplay between CFTR and pendrin: Physiological and pathophysiological relevance. *Front. Biosci.* **27**, 75 (2022).

50. S. M. Wall, The role of pendrin in blood pressure regulation. *Am. J. Physiol. Renal Physiol.* **310**, F193–F203 (2016).

51. J. B. Dacks, M. S. Robinson, Outerwear through the ages: Evolutionary cell biology of vesicle coats. *Curr. Opin. Cell Biol.* **47**, 108–116 (2017).

52. G. A. Mardones, P. V. Burgos, Y. Lin, D. P. Kloer, J. G. Magadan, J. H. Hurley, J. S. Bonifacino, Structural basis for the recognition of tyrosine-based sorting signals by the μ3A subunit of the AP-3 adaptor complex. *J. Biol. Chem.* **288**, 9563–9571 (2013).

53. C. M. Guardia, R. De Pace, R. Mattera, J. S. Bonifacino, Neuronal functions of adaptor complexes involved in protein sorting. *Curr. Opin. Neurobiol.* **51**, 103–110 (2018).

54. R. Mattera, G. G. Farias, G. A. Mardones, J. S. Bonifacino, Co-assembly of viral envelope glycoproteins regulates their polarized sorting in neurons. *PLOS Pathog.* **10**, e1004107 (2014).

55. K. Honda, H. J. Lee, A. J. Griffith, I. Roux, Dissection of the endolymphatic sac from mice. *J. Vis. Exp.* **29**, e62375 (2021).

56. J. D. Thompson, D. G. Higgins, T. J. Gibson, CLUSTAL W: Improving the sensitivity of progressive multiple sequence alignment through sequence weighting, position-specific gap penalties and weight matrix choice. *Nucleic Acids Res.* **22**, 4673–4680 (1994).

57. E. Lindahl, B. Hess, D. Van, Der Spoel, GROMACS 3.0: A package for molecular simulation and trajectory analysis. *J. Mol. Model.* **7**, 306–317 (2001).

58. X. Daura, K. Gademann, B. Jaun, D. Seebach, W. F. Van Gunsteren, A. E. Mark, Peptide folding: When simulation meets experiment. *Angew. Chem. Int. Ed. Engl.* **38**, 236–240 (1999).

59. I. A. Belyantseva, Helios® Gene Gun-mediated transfection of the inner ear sensory epithelium: Recent updates. *Methods Mol. Biol.* **1427**, 3–26 (2016).

60. S. J. Lord, K. B. Velle, R. D. Mullins, L. K. Fritz-Laylin, SuperPlots: Communicating reproducibility and variability in cell biology. *J. Cell Biol.* **219**, e202001064 (2020).

61. R. L. Miller, P. Zhang, M. Smith, V. Beaulieu, T. G. Paunescu, D. Brown, S. Breton, R. D. Nelson, V-ATPase B1-subunit promoter drives expression of EGFP in intercalated cells of kidney, clear cells of epididymis and airway cells of lung in transgenic mice. *Am. J. Physiol. Cell Physiol.* **288**, C1134–C1144 (2005).

62. S. J. Tunster, Genetic sex determination of mice by simplex PCR. *Biol. Sex Differ.* **8**, 31 (2017).

63. A. B. Vojtek, S. M. Hollenberg, Ras-Raf interaction: Two-hybrid analysis. *Methods Enzymol.* **255**, 331–342 (1995).

64. P. Bartel, C.-T. Chien, R. Sternglanz, S. Fields, "Using the two-hybrid system to detect protein-protein interactions" in *Cellular Interactions in Development: A Practical Approach*, D. A. Hartley, Ed. (Oxford Univ. Press, 1993), pp. 153–179.

65. M. Fromont-Racine, J. C. Rain, P. Legrain, Toward a functional analysis of the yeast genome through exhaustive two-hybrid screens. *Nat. Genet.* **16**, 277–282 (1997).

66. E. Formstecher, S. Aresta, V. Collura, A. Hamburger, A. Meil, A. Trehin, C. Reverdy, V. Betin, S. Maire, C. Brun, B. Jacq, M. Arpin, Y. Bellaiche, S. Bellusci, P. Benaroch, M. Bornens, R. Chanet, P. Chavrier, O. Delattre, V. Doye, R. Fehon, G. Faye, T. Galli, J. A. Girault, B. Goud, J. de Gunzburg, L. Johannes, M. P. Junier, V. Mirouse, A. Mukherjee, D. Papadopoulo, F. Perez, A. Plessis, C. Rosse, S. Saule, D. Stoppa-Lyonnet, A. Vincent, M. White, P. Legrain, J. Wojcik, J. Camonis, L. Daviet, Protein interaction mapping: A *Drosophila* case study. *Genome Res.* **15**, 376–384 (2005).

67. F. Colland, X. Jacq, V. Trouplin, C. Mougin, C. Groizeleau, A. Hamburger, A. Meil, J. Wojcik, P. Legrain, J. M. Gauthier, Functional proteomics mapping of a human signaling pathway. *Genome Res.* **14**, 1324–1332 (2004).

68. L. Lin, R. S. Petralia, R. Lake, Y. X. Wang, D. A. Hoffman, A novel structure associated with aging is augmented in the DPP6-KO mouse brain. *Acta Neuropathol. Commun.* **8**, 197 (2020).

69. J. Li, W. Han, K. A. Pelkey, J. Duan, X. Mao, Y. X. Wang, M. T. Craig, L. Dong, R. S. Petralia, C. J. McBain, W. Lu, Molecular dissection of neuroligin 2 and Slitrk3 reveals an essential framework for GABAergic synapse development. *Neuron* **96**, 808–826.e8 (2017).

70. W. Han, J. Li, K. A. Pelkey, S. Pandey, X. Chen, Y. X. Wang, K. Wu, L. Ge, T. Li, D. Castellano, C. Liu, L. G. Wu, R. S. Petralia, J. W. Lynch, C. J. McBain, W. Lu, Shisa7 is a $GABA_A$ receptor auxiliary subunit controlling benzodiazepine actions. *Science* **366**, 246–250 (2019).

71. G. K. Varshney, W. Pei, M. C. LaFave, J. Idol, L. Xu, V. Gallardo, B. Carrington, K. Bishop, M. Jones, M. Li, U. Harper, S. C. Huang, A. Prakash, W. Chen, R. Sood, J. Ledin, S. M. Burgess, High-throughput gene targeting and phenotyping in zebrafish using CRISPR/Cas9. *Genome Res.* **25**, 1030–1042 (2015).

72. H. Wang, H. Yang, C. S. Shivalila, M. M. Dawlaty, A. W. Cheng, F. Zhang, R. Jaenisch, One-step generation of mice carrying mutations in multiple genes by CRISPR/Cas-mediated genome engineering. *Cell* **153**, 910–918 (2013).

73. E. M. Keithley, C. Canto, Q. Y. Zheng, N. Fischel-Ghodsian, K. R. Johnson, Age-related hearing loss and the ahl locus in mice. *Hear. Res.* **188**, 21–28 (2004).

74. K. R. Johnson, L. C. Erway, S. A. Cook, J. F. Willott, Q. Y. Zheng, A major gene affecting age-related hearing loss in C57BL/6J mice. *Hear. Res.* **114**, 83–92 (1997).

75. C. Bassot, G. Minervini, E. Leonardi, S. C. Tosatto, Mapping pathogenic mutations suggests an innovative structural model for the pendrin (SLC26A4) transmembrane domain. *Biochimie* **132**, 109–120 (2017).
